# Comprehensive Identification and Mechanistic Evaluation of Novel DHODH Inhibitors as Potent Broad-Spectrum Antiviral Agents

**DOI:** 10.3390/ph18091416

**Published:** 2025-09-20

**Authors:** Chao Zhang, Shiyang Sun, Huiru Xie, Yongzhao Ding, Chun Hu, Jialin Guo, Junhai Xiao

**Affiliations:** 1 State Key Laboratory of National Security Specially Needed Medicines, Beijing 100039, China; zhangchaoylh@126.com (C.Z.); noah97sun@163.com (S.S.); xiehuiru0808@163.com (H.X.); 13308109105@163.com (Y.D.); 2 Key Laboratory of Structure-Based Drug Design Discovery, Ministry of Education, School of Pharmaceutical Engineering, Shenyang Pharmaceutical University, Shenyang 110016, China; chunhu@syphu.edu.cn

**Keywords:** dihydroorotate dehydrogenase, broad-spectrum antiviral agents, MD simulation, influenza virus, SARS-CoV-2

## Abstract

**Background/Objectives**: This study identifies novel dihydroorotate dehydrogenase (DHODH) inhibitors exhibiting potent broad-spectrum antiviral agents, particularly against influenza A virus (A/PR/8/34(H1N1)) and SARS-CoV-2. **Methods**: Structure-based virtual screening of 1.6 million compounds (ChemDiv and TargetMol databases) yielded 10 candidates, with compounds **6**, **9**, and **10** demonstrating significant anti-influenza activity (IC_50_ = 4.85 ± 0.58, 7.35 ± 1.65, and 1.75 ± 0.28 μM, respectively). Building on these, molecular hybridization principles and scaffold hopping principles were applied to design and synthesize six novel compounds (**11**–**16**) through cyclization, coupling, and carboxylate deprotection. Prior to subsequent biological assays, the molecular structures of each compound were elucidated by NMR spectroscopy and MS. Their antiviral activities were subsequently assessed against both influenza virus and SARS-CoV-2. The compound **11**, demonstrating the most potent antiviral activity, was further subjected to surface plasmon resonance (SPR) analysis to assess its binding affinity for human DHODH. **Results**: Compound **11** emerged as the most potent DHODH inhibitor (*K*_D_ = 6.06 μM), exhibiting superior broad-spectrum antiviral activities (IC_50_ = 0.85 ± 0.05 μM, A/PR/8/34(H1N1); IC_50_ = 3.60 ± 0.67 μM, SARS-CoV-2) to the reported DHODH inhibitor (Teriflunomide**,** IC_50_ = 35.02 ± 3.33 μM, A/PR/8/34(H1N1); IC_50_ = 26.06 ± 4.32 μM, SARS-CoV-2). Mechanistic evaluations via 100 ns MD simulations and QM/MM calculations revealed stable binding interactions, particularly hydrogen bonds with GLN47 and ARG136, while alanine scanning mutagenesis confirmed these residues’ critical roles in binding stability. **Conclusions**: This work identifies compound **11** as a potent broad-spectrum antiviral compound, offering a promising strategy for broad-spectrum antiviral therapy against RNA viruses by depleting pyrimidine pools essential for viral replication.

## 1. Introduction

The global landscape of viral infections continued to pose significant public health threats due to emerging and re-emerging pathogens—such as influenza, SARS-CoV-2, Ebola, dengue (DENV), Zika (ZIKV), and Chikungunya viruses—which often exhibited high mutation rates and transmissibility, straining healthcare systems worldwide [1]. Current treatments, including virus-specific direct-acting antivirals and supportive care, remain limited by narrow-spectrum activity, drug resistance, and inadequate efficacy against genetically diverse viruses. While some broad-spectrum antiviral candidates exist, many suffered from poorly defined targets or suboptimal in vivo performance. Thus, there was a pressing demand for the development of novel antivirals that target conserved host or viral mechanisms, such as dihydroorotate dehydrogenase (DHODH), a key enzyme in pyrimidine biosynthesis utilized by multiple RNA viruses for replication [1].

DHODH was an integral flavoprotein anchored to the inner mitochondrial membrane. It catalyzed the oxidation of dihydroorotate (DHO) to orotate (ORO), which was the fourth and rate-limiting reaction in de novo pyrimidine synthesis. This redox reaction concurrently channeled electrons into the mitochondrial respiratory chain via the ubiquinone pool [1]. Structurally, human DHODH comprised an *N*-terminal α-helical membrane-anchoring domain and a *C*-terminal α/*β*-barrel domain that forms the catalytic site for FMN and dihydroorotate binding [2,3,4].

DHODH inhibitors had been successfully repurposed for autoimmune disorders. Leflunomide, approved for rheumatoid arthritis (1998), was metabolized to teriflunomide (A771726), which inhibited DHODH with an IC_50_ of 307 nM by competing with ubiquinone [5]. Teriflunomide received FDA approval for multiple sclerosis (2012) due to its ability to suppress T-cell proliferation through pyrimidine depletion [6]. Brequinar advanced to Phase II cancer trials but was limited by hematological toxicity (CC_50_ > 5 μM) [7]. Recent candidates like vidofludimus (IMU-838) and BAY 2402234 exhibited enhanced selectivity (IC_50_ = 41–160 nM) and were in Phase II trials for ulcerative colitis and acute myeloid leukemia [8].

**Figure 1:**
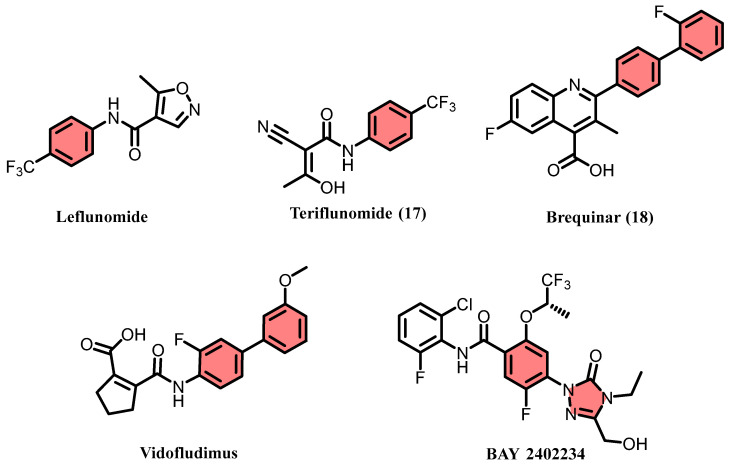
The reported DHODH inhibitors.

**Figure 2:**
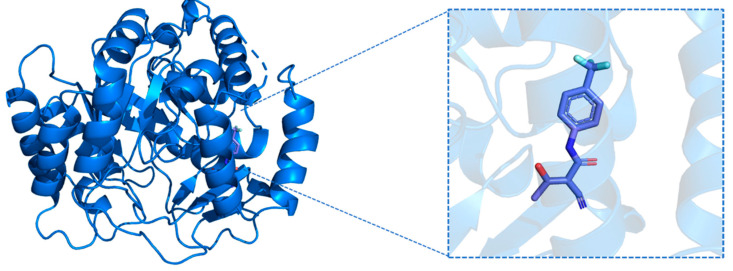
Crystal structure of DHODH in complex with **17** (PDB ID: 1D3H).

DHODH inhibition depleted pyrimidine nucleotides, disrupting viral RNA synthesis. Thus, DHODH inhibitors were potent broad-spectrum antiviral compounds. Brequinar (Figure 1) inhibited DENV and ZIKV viruses (EC_50_ = 17–61 nM) by starving viral polymerases of nucleotides [9]. BAY 2,402,234 reduced viral replication in human lung organoids (EC_50_ = 32 nM) and mitigated cytokine storms by downregulating IL-6 [10]. Coadministration with molnupiravir enhanced antiviral efficacy >1000-fold against SARS-CoV-2 by dual targeting of pyrimidine synthesis and viral RNA elongation [11]. Thus, DHODH inhibitors represented a promising strategy for antiviral therapy, particularly against RNA viruses dependent on host pyrimidine pools [11]. Key challenges were optimizing blood–brain barrier penetration (e.g., for neurotropic viruses) and minimizing hematological toxicity via prodrug designs [12]. The multifunctional nature of DHODH inhibitors, which encompassed the suppression of viral replication, upregulation of interferon-stimulated genes (ISGs), and mitigation of cytokine storms, positioned them as a powerful therapeutic approach for combating viral infections [13]. There had been multiple reported on the crystal structures of DHODH and teriflunomide (PDB ID: 1D3H, Figure 2), which provide reference for designing new DHODH inhibitors. Therefore, developing inhibitors based on DHODH is an important means of developing broad-spectrum antiviral drugs.

**Figure 3:**
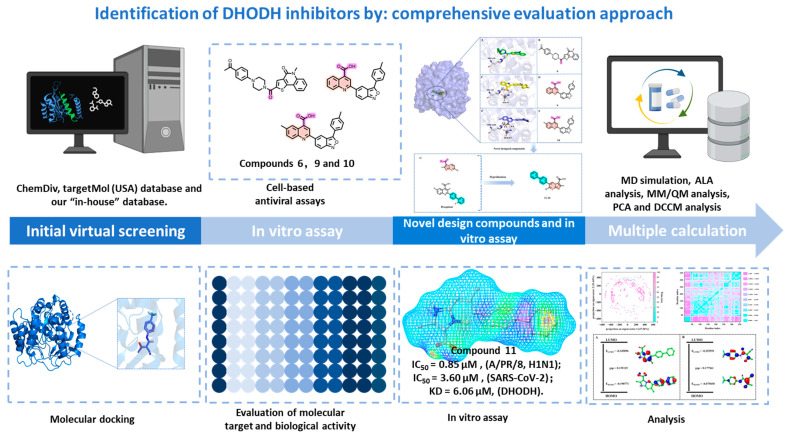
The flow of the multi-step procedure in this study.

This study employed an integrated computational and experimental strategy, utilizing molecular docking to target the active site occupied by compound **17**, followed by molecular dynamics (MD) simulations, ADMET profiling, in vitro cell-based assays, and quantum mechanics/molecular mechanics (QM/MM) calculations to discover and characterize novel DHODH inhibitors from the 1.6 million compounds sourced from the ChemDiv and TargetMol (USA) databases. Following initial screening for favorable interactions with the DHODH active site and acceptable ADMET properties, lead compounds were identified. Their binding affinities and complex stabilities with DHODH were subsequently assessed via 20 ns MD simulations to estimate binding free energies. Finally, the most promising candidates (compounds **1**–**10**) were progressed to experimental validation in cell-based assays. Subsequently, we designed and synthesized six compounds with an imidazo[1,2-*a*]pyridine-3-carboxylic acid scaffold which were subjected to evaluate their antiviral activity. A surface plasmon resonance (SPR) experiment was used to identify DHODH inhibitors with better binding affinities. MD simulations spanning 100 ns and QM/MM calculations were conducted on candidate compounds exhibiting potency comparable to or greater than the reference molecule **17**. These analyses were anticipated to elucidate the precise binding mechanisms and key electronic properties, thereby establishing a robust foundation for the development of novel DHODH inhibitors with broad-spectrum antiviral efficacy. A schematic diagram outlining the flow of the multi-step procedure was presented in Figure 3. Ultimately, we identified compound **11** as a novel DHODH inhibitor exhibiting broad-spectrum antiviral efficacy. The distinct architecture of this scaffold serves as a strategic framework that directs subsequent drug discovery and molecular refinement efforts.

## 2. Results

### 2.1. Virtual Screening

To validate the docking protocol, the cognate ligand (teriflunomide, **17**, Figure 1) was re-docked into the binding site of its human DHODH co-crystal structure (PDB: 1D3H). The top-ranked pose demonstrated a near-perfect spatial overlap with the experimental conformation, yielding a remarkably low RMSD of 0.384 Å (Figure S1), which confirmed the accuracy of the docking setup. A systematic structure-based virtual screening campaign computationally profiled 1.6 million compounds from the ChemDiv and TargetMol (USA) databases, culminating in the identification of 200 high-priority candidate molecules. The selected 200 compounds were refined using threshold criteria of Glide docking score ≤ −9 kcal/mol and Molecular Mechanics/Generalized Born Surface Area (MM/GBSA) binding free energy ≤ −50 kcal/mol, which were similar with the reference compound **17** (docking score = −9.100 kcal/mol and MM/GBSA binding free energy = −44.01 kcal/mol). Then, we yielded a subset of 10 prioritized candidates with betted docking scores and lower MM/GBSA binding free energy than the reference compound **17** (Table 1). These compounds underwent 20 ns MD simulations alongside reference compound **17** (Figure S2). Post-equilibration, most compounds exhibited converged RMSD profiles, indicating stable binding-mode retention throughout simulations (Figure S2). Notably, all candidates demonstrated more favorable MM/GBSA binding free energies than compound **17**, signifying superior binding affinity and inhibitory potential (Figure S2 and Table S1).

ADMET property assessment was conducted to prioritize compounds with optimal pharmacokinetic profiles [14] relative to reference standards teriflunomide (**17**) and brequinar (**18**) (Tables S1–S3). Candidates exhibited moderate-to-high aqueous solubility (levels 1–2), with compounds **1**, **9**, and **10** displaying the lowest solubility while others demonstrated significantly greater solubility than brequinar (**18**). Superior intestinal absorption (level 2) was observed for compounds **3**–**8**, outperforming **1** (level 3), **2**, **9**, and **10** (all level 1). Toxicity screening predicted no Ames mutagenicity for any candidate. Chronic rodent toxicity (oral LD_50_) assessments revealed favorable profiles for **2** (3.67 g/kg) and **3** (8.33 g/kg) compared to **17** (1.50 g/kg). Collectively, candidates exhibited enhanced pharmacokinetic properties and reduced toxicity risks relative to reference inhibitors.

### 2.2. Biological Activity Assessment of the Candidate Compounds

DHODH inhibitors demonstrated potent anti-influenza activity through disruption of host pyrimidine biosynthesis, thereby suppressing viral replication [15]. The antiviral potency of the ten compounds was assessed in vitro against the influenza A/PR/8/34(H1N1) strain, utilizing compound **17** as a reference compound for comparison. Antiviral potency (IC_50_), cytotoxicity (CC_50_), and selectivity indices (SI = CC_50_/IC_50_) were determined to quantify therapeutic potential (Table 1). Compounds **1**–**10** were purchased from TargetMol Chemicals Inc. (Boston, MA, USA). The MDCK cells, short for Madin–Darby Canine Kidney cells, were used for influenza infection. Compounds **6**, **9**, and **10** exhibited the strongest inhibition with IC_50_ values of 4.85 ± 0.58, 7.35 ± 1.65, and 1.75 ± 0.28 μM, respectively (Table 1). Cytotoxic profiles varied significantly: compound **1** demonstrated the highest toxicity (CC_50_ = 34.52 μM in MDCK cells), while compounds **2**–**10** showed substantially lower cytotoxicity (CC_50_ >200 μM). The calculated SI values reflect each compound’s therapeutic window, with compounds **6**, **9**, and **10** exhibiting superior selectivity profiles relative to compound **17**.

Generally, compounds **6** (IC_50_ = 4.85 μM), **9** (IC_50_ = 7.35 μM), and **10** (IC_50_ = 1.75 μM) demonstrated potent antiviral activity against influenza A virus (A/PR/8/34(H1N1)), establishing critical structure–activity benchmarks for rational inhibitor design.

### 2.3. Design of Novel Compounds

**Figure 4:**
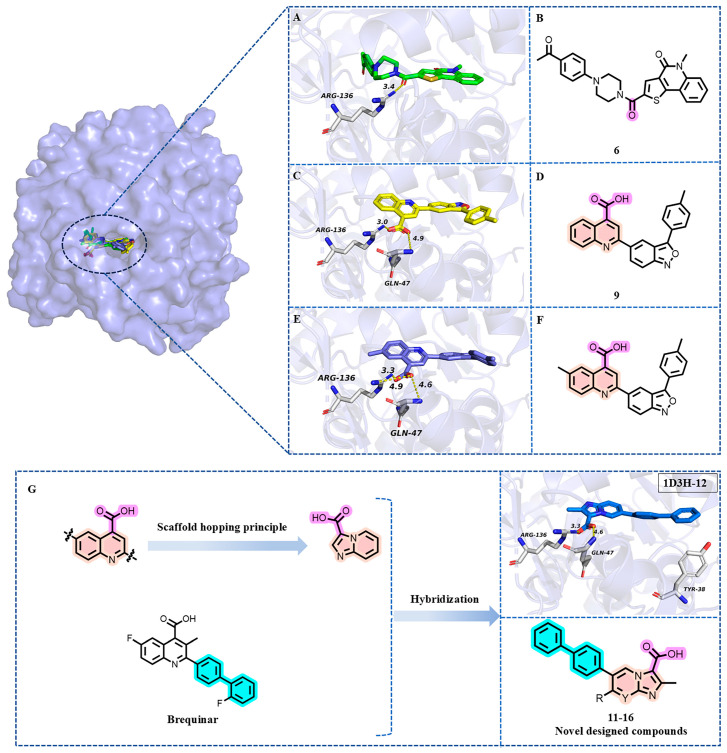
The docking poses of 1D3H-**6** (**A**), 1D3H-**9** (**C**), and 1D3H-**10** (**E**). The H-bond interaction was labeled as the yellow dash line. The structures of **6** (**B**), **9** (**D**), and **10** (**F**). The design of novel designed compounds (**G**).

Molecular docking revealed that compounds **6**, **9**, and **10** formed hydrogen bonds with key residues GLN47 and ARG136, suggesting a critical mechanism for binding affinity and antiviral activity. This analysis identified the carbonyl oxygen in **6** and the quinoline carboxylic acid scaffold in **9/10** as essential pharmacophoric elements (Figure 4A–F). Since no significant interactions were observed between the aromatic π-system of the compounds and the protein, the quinoline carboxylic acid scaffold was replaced with an imidazo[1,2-*a*]pyridine-3-carboxylic acid scaffold [16,17,18] using the scaffold hopping principle—a privileged structural motif found in anti-infective agents. Brequinar (**18**), a DHODH inhibitor, had been reported to possess broad-spectrum antiviral efficacy [15]. Accordingly, we selected the imidazo[1,2-*a*]pyridine-3-carboxylic acid scaffold as the core structure and incorporated the biphenyl fragment from brequinar’s architecture. Using the molecular hybridization approach, we designed six target compounds (Figure 4G). According to the molecular docking study, compound **12** could generate hydrogen bonds with GLN47 and ARG136. In addition, the benzene ring in the biphenyl structure could form a π–π interaction with TYR38 (Figure 4G). Consequently, the newly designed compound **12**, featuring the imidazo[1,2-*a*]pyridine-3-carboxylic acid scaffold, not only retained the critical interactions observed in compounds **9**/**10** but also established novel interactions with residue TYR38. Furthermore, compound **12** exhibited a docking score of −13.407 kcal/mol and an MM/GBSA binding free energy of −70.36 kcal/mol, which were comparable to or even superior to those of compounds **9**/**10**. These computational results demonstrated the rationality of designing this type of target compound.

**Scheme 1:**
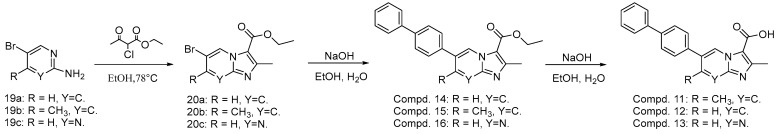
Schematic diagram of the synthetic route of the key compounds **11**–**16**.

Subsequently, we synthesized six novel target compounds, **14**–**16,** which were synthesized via cyclization and cross-coupling reactions, while **11**–**13** were prepared through sequential cyclization, coupling, and carboxylate deprotection (Scheme 1). Further spectral details are available in the Supplementary Materials (Table S4).

### 2.4. Biological Activity Assessment of the Novel Designed Compounds

DHODH inhibitors demonstrated broad-spectrum antiviral activity, exhibiting efficacy against both influenza viruses and SARS-CoV-2 [15]. A Vero-ACE2-TMPRSS2 (labelled ‘Vero’ hereafter) cell line was used for SARS-CoV-2. The antiviral profiles of candidate compounds **11**–**16** and reference compound **17** were characterized through determination of IC_50_ values against influenza A (A/PR/8/34(H1N1)) and SARS-CoV-2, cytotoxicity (CC_50_), and selectivity indices (SI = CC_50_/IC_50_) (Table 2). Compound **11** emerged as the most potent candidate, demonstrating exceptional anti-influenza activity (IC_50_ = 0.85 ± 0.05 μM) and significant SARS-CoV-2 inhibition (IC_50_ = 3.60 ± 0.67 μM) (Table 2). This dual activity surpassed both compound **17** (influenza IC_50_ = 35.02 ± 3.33 μM; SARS-CoV-2 IC_50_ = 26.06 ± 4.32 μM) and remdesivir (SARS-CoV-2 IC_50_ = 4.95 ± 0.78 μM). All derivatives (**11**–**16**) exhibited minimal cytotoxicity (CC_50_ > 200 μM) in MDCK and Vero cells, resulting in favorable therapeutic windows as quantified by SI values (Table 2). Baloxavir marboxil (IC_50_ = 9.67 nM), a recognized inhibitor of influenza virus replication, was selected as the reference compound for the influenza assays. Remdesivir (IC_50_ = 4.95 μM) was used as the reference compound for the SARS-CoV-2 experiments. Although compound **11** exhibited weaker inhibitory activity against influenza virus than the established antiviral baloxavir marboxil (IC_50_ = 9.67 ± 1.12 nM), it demonstrated superior efficacy against SARS-CoV-2 compared to remdesivir (IC_50_ = 4.95 ± 0.78 μM). While the values are comparable, it is important to acknowledge that they act via completely different mechanisms. Generally, **11** was the most potent antiviral compound against influenza A virus (IC_50_ = 0.85 ± 0.05 μM, A/PR/8/34(H1N1)) and SARS-CoV-2 (IC_50_ = 3.60 ± 0.67 μM).

The structure–activity relationship (SAR) analysis is summarized as follows: The carboxylic acid group was identified as a critical moiety for antiviral activity. For instance, hydrolysis of the ester group in compound **15** to yield the carboxylic acid derivative (compound **11**) resulted in significantly enhanced antiviral potency. Furthermore, compounds containing the imidazo[1,2-*a*]pyrimidine scaffold (e.g., compound **13**) demonstrated superior anti-influenza virus activity compared to those with an imidazo[1,2-*a*]pyridine core (e.g., compound **12**), suggesting that the imidazo[1,2-*a*]pyrimidine ring is a more favorable structural framework. Additionally, the markedly higher antiviral activity of compound **11** relative to compound **12** may be attributed to the key influence of the methyl group at the 7-position of the imidazo[1,2-*a*]pyridine skeleton. In conclusion, these SAR insights provide valuable guidance for the future design of optimized compounds.

### 2.5. Validation of Molecular Target for Selected Compounds

To verify the binding of candidate molecules to DHODH, we employed SPR to assess the binding affinities of compounds **11**, **17**, and **18**. Compound **11** exhibited a higher binding affinity (*K*_D_ = 6.06 μM, Table 3) compared to compounds **17** (*K*_D_ = 23.9 μM, Table 3) and **18** (*K*_D_ = 6.13 μM, Table 3). Consequently, compound **11** was identified as a potent inhibitor of DHODH.

### 2.6. MD Simulation Analysis

MD simulations characterized the structural stability and conformational dynamics of protein–ligand complexes for selected DHODH inhibitors. Following integrated computational and pharmacological assessment, complexes of DHODH with compounds **11**, **17**, and **18** underwent 100 ns all-atom MD simulations to evaluate binding stability and ensemble behavior, elucidating mechanistic insights into inhibitory efficacy. Trajectory analysis revealed convergent root-mean-square deviation (RMSD) profiles across replicate simulations, confirming methodological robustness and trajectory convergence.

#### 2.6.1. Stability of Dynamics Trajectory from RMSD Analysis

**Figure 5:**
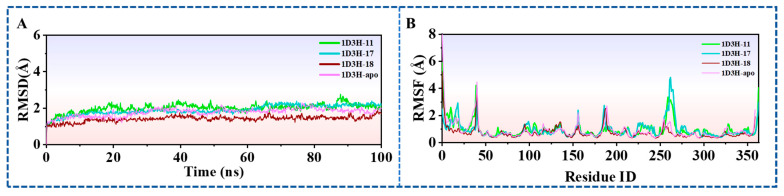
The RMSD (**A**) and RMSF (**B**) of protein backbone atoms monitored during the 100 ns MD simulations.

The structural stability of each complex throughout the 100 ns production run was quantified by their RMSD profiles, as depicted in Figure 5. The average RMSD values of **11**, **17**, and **18** ranged from 1.5 to 2.0 Å. Obviously, all complexes reached equilibrium and exhibited sufficient stability for further analysis.

#### 2.6.2. Analysis of Residue-Specific Mobility Derived from Root-Mean-Square Fluctuation (RMSF) Data

Residue-specific conformational dynamics govern the stability of each complex. RMSF analysis quantifies positional variance of individual amino acids relative to their time-averaged positions, providing critical insights into regional structural flexibility and residue-specific contributions to complex stability. Elevated RMSF values indicated enhanced atomic mobility within protein segments, while reduced values reflected conformational rigidity. As illustrated in Figure 5, all three complexes (**11**, **17**, **18**) exhibited highly congruent residue fluctuation patterns throughout 100 ns simulations. This convergence indicates that each ligand establishes stabilizing interactions with DHODH protein, effectively constraining conformational flexibility across the binding interface.

#### 2.6.3. Analysis of Intermolecular Interactions Throughout the 100 ns MD Simulation

**Figure 6:**
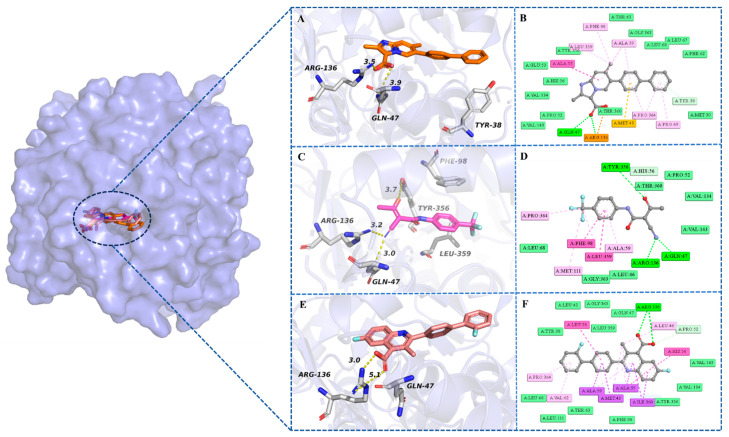
The docking poses of 1D3H-**11** (**A**,**B**), 1D3H-**17** (**C**,**D**), and 1D3H-**18** (**E**,**F**). The H-bond interaction was labeled as the yellow dash line.

**Figure 7:**
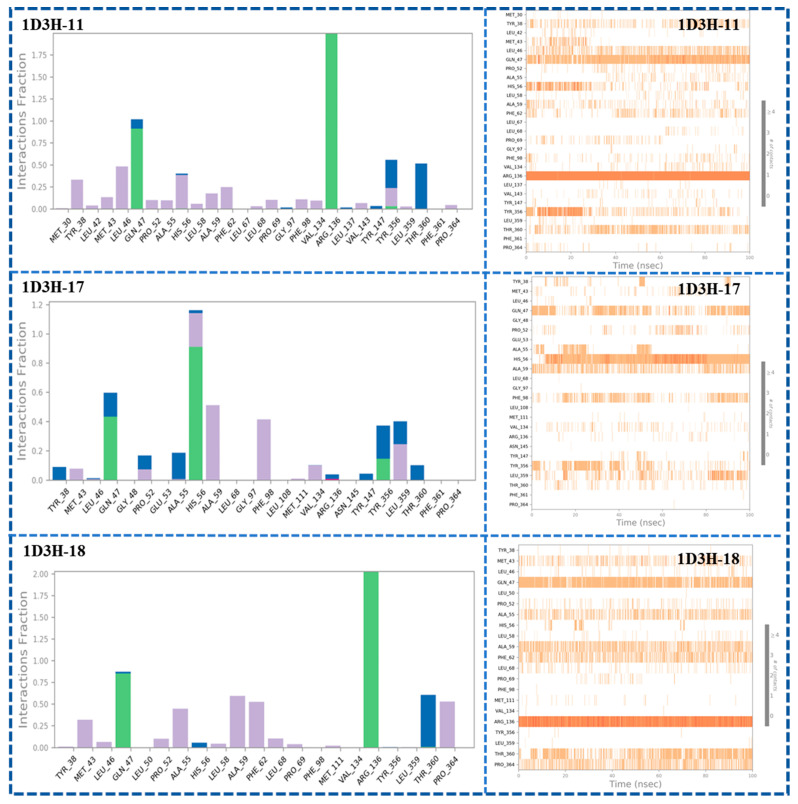
Protein–ligand contacts of the complexes (1D3H-**11**, 1D3H-**17**, and 1D3H-**18**) during the 100 ns simulation. The abscissa of the cumulated histogram indicates the vital residues that participate in the interactions, while the ordinate denotes interactions percentage. The interactions fraction is calculated depending on their duration percentage along the whole 100 ns MD simulations trajectory.

Molecular docking revealed consistent hydrogen bonding interactions between all three ligands and residue GLN47 (Figure 6). Subsequent 100 ns MD simulations enabled rigorous characterization of binding modes, quantifying four interaction types: hydrogen bonds, hydrophobic contacts, ionic bridges, and water-mediated interactions. Hydrophobic contacts involving GLN47 were consistently maintained throughout the simulation in all complexes. Additionally, this residue formed hydrogen bonds with compounds **11** and **18**, an interaction network that also included ARG136. Compound **17** engaged GLN47, HIS56, and TYR356 through hydrogen bonds (Figure 7). Interaction frequency heatmaps (darker hues indicating higher occurrence) demonstrated persistent involvement of catalytic site residues GLN47, HIS56, and TYR356 throughout the simulations. Notably, GLN47 maintained stable ligand distances during the 100 ns trajectory, confirming its critical role in molecular recognition.

#### 2.6.4. Ligands Properties Analysis Thorough the 100 ns MD Simulation

**Figure 8:**
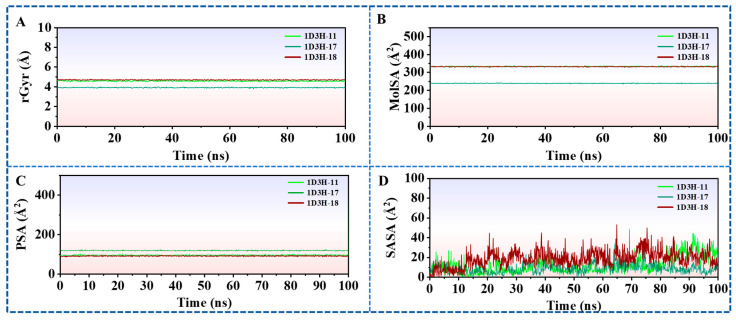
Ligand properties ((**A**), rGyr; (**B**), MolSA; (**C**), PSA; (**D**), SASA) during the 100 ns MD simulations.

Critical for characterizing protein–ligand binding modes, the trajectory-derived descriptors—including radius of gyration (rGyr), molecular surface area (MolSA), solvent-accessible surface area (SASA), and polar surface area (PSA)—provide key insights into molecular recognition. rGyr values averaging ~5 Å (Figure 8A) indicated substantial active site volume, suggesting opportunities for strategic functional group incorporation to enhance binding affinity. MolSA analysis (corresponding to van der Waals surface area) revealed consistent molecular dimensions across compounds **11**, **17**, and **18** (Figure 8B). PSA and SASA profiles (Figure 8C,D) aligned with hydrogen-bonding patterns and solvation effects observed in docking and MD simulations. Collectively, these parameters provide structural blueprints for rational optimization of DHODH inhibitors.

### 2.7. Alanine Scanning Mutagenesis (ASM)

**Figure 9:**
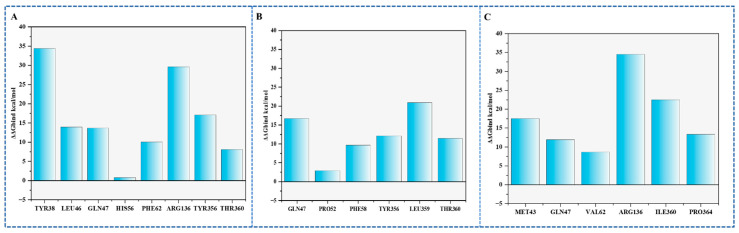
The key amino acids in complexes 1D3H-**11** (**A**), 1D3H-**17** (**B**), and 1D3H-**18** (**C**) analyzed by ASM analysis.

ASM was employed to further validate the explored binding mode. Systematic mutation of key residues (TYR38, LEU46, GLN47, HIS56, PHE62, ARG136, TYR356, and THR360) in the complex 1D3H-**11** significantly compromised structural stability, as evidenced by a substantial increase in the computed binding free energy change (ΔΔG_bind_) for each variant (Figure 9). Similarly, mutating the important amino acid residues (GLN47, PRO52, PHE58, TYR356, LEU359, and THR360) of complex 1D3H-**17** into alanine all resulted in the increased ΔΔG_bind_ values (Figure 9). Obviously, mutating the important amino acid residues of complex 1D3H-**18** into alanine also resulted in the increased ΔΔG_bind_ values (Figure 9). Consequently, these amino acid side chains were instrumental in mediating the protein–ligand association.

ASM validated critical binding site residues by systematically substituting key amino acids in DHODH complexes. Mutations of TYR38, LEU46, GLN47, HIS56, PHE62, ARG136, TYR356, and THR360 in the complex 1D3H-**11** significantly destabilized binding (ΔΔG_bind_ > 0 kcal/mol, Figure 9). Analogous alanine substitutions at GLN47, PRO52, PHE58, TYR356, LEU359, and THR360 in the complex 1D3H-**17** similarly increased ΔΔG_bind_ values. Equivalent mutagenesis of essential residues in the complex 1D3H-**18** likewise impaired binding affinity. These results collectively demonstrate that side-chain functionalities at these positions are essential for productive ligand recognition and complex stabilization. Furthermore, SPR affinity assay results demonstrated that compounds **11** and **18** exhibited superior binding affinity to the DHODH protein compared to compound **17**, which was consistent with the ASM experimental findings. The key amino acid residues in 1D3H-**11** and 1D3H-**18** likely played more significant roles in mediating the interaction with DHODH (Figure 9) than 1D3H-**17**.

### 2.8. QM/MM Calculations

**Figure 10:**
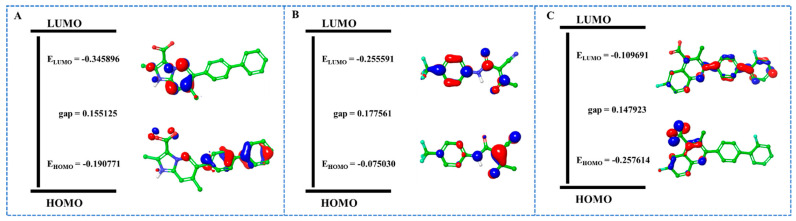
HOMO and LUMO analysis of **11** (**A**), **17** (**B**), and **18** (**C**) using QM/MM analysis.

To characterize the π–π stacking and electrostatic forces within the system, the highest occupied molecular orbital (HOMO) and the lowest unoccupied molecular orbital (LUMO) were computed. These frontier molecular orbitals are critically involved in facilitating interactions between the ligands and amino acid residues. Notably, both the HOMO and LUMO were found to be spatially distributed primarily over the aromatic moieties in all three compounds (Figure 10). This localization aligns with the π–π stacking and electrostatic interactions identified through molecular docking and molecular dynamics simulations, thereby offering a coherent mechanistic interpretation of the binding events and informing the rational design of DHODH inhibitors.

### 2.9. Principal Component Analysis (PCA) and Dynamic Cross-Correlation Matrices (DCCM) Analysis

**Figure 11:**
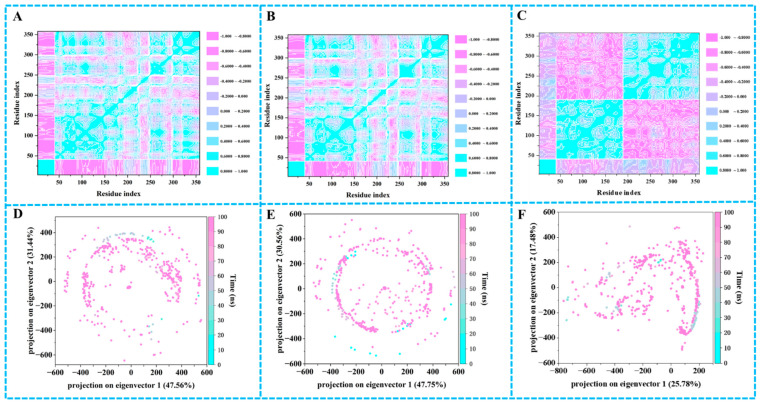
DCCM analysis for 1D3H-**11** (**A**), 1D3H-**17** (**B**), and 1D3H-**18** (**C**). Residues exhibiting strongly correlated motions within the DHODH structure were indicated by high positive values (cyan), whereas those with strongly anti-correlated motions were represented by negative values (purple). PCA analysis for 1D3H-**11** (**D**), 1D3H-**17** (**E**), and 1D3H-**18** (**F**).

DCCM analysis characterized residue motion coupling in complexes of **11**, **17**, and **18** during MD simulations (Figure 11). Correlation coefficients (range: −1 to +1) revealed concerted motions (positive values, cyan) and anti-correlated displacements (negative values, purple). Enhanced diagonal correlation signatures indicated pronounced collective dynamics within these complexes, suggesting ligand-mediated stabilization of protein conformational ensembles.

PCA characterized conformational landscapes of protein–ligand complexes during MD simulations. Projections of trajectories onto the PC1-PC2 subspace revealed restricted phase space occupancy for complexes **11**, **17**, and **18** (Figure 11), indicating limited structural flexibility. This constrained sampling suggested rigid binding modes that may enhance complex stability through reduced entropic penalties.

## 3. Discussion

The present study identifies compound **11** as a potent dual inhibitor of influenza A (H1N1) and SARS-CoV-2 through targeted inhibition of DHODH. Its superior binding affinity and antiviral efficacy over reference inhibitor teriflunomide (**17**) necessitate a critical examination of its structural and mechanistic uniqueness. Unlike classical DHODH inhibitors, compound **11** exploits a distinct hydrophobic subpocket via π–π stacking with TYR38, complementing conserved hydrogen bonds with GLN47 and ARG136. This extended interaction network was validated by molecular dynamics and enhanced antiviral potency.

The SAR reveals that subtle modifications significantly alter efficacy. For instance, replacing the pyridine ring in compound **12** with a pyrimidine (compound **13**) enhanced H-bond acceptor capacity, reducing IC_50_ from >100 μM to 1.08 μM. Conversely, esterification (compound **15**) abolished activity due to lost H-bond donation to ARG136. These findings underscore the criticality of hydrogen bond donors/acceptors and scaffold rigidity in maintaining pharmacophore integrity.

Beyond antiviral effects, DHODH inhibition imposes immunomodulatory outcomes via pyrimidine depletion. Compound **11**’s dual action might synergistically suppress replication and attenuate hyperinflammation. This duality necessitates careful in vivo evaluation of immune cell profiling. However, prolonged immunosuppression risks impairing viral clearance in immunocompromised hosts.

Computational data showed strong convergence but also critical nuances. MD simulations confirmed stable binding to key residues GLN47 and ARG136 (Figure 5, Figure 6, Figure 7 and Figure 8). QM/MM calculations revealed π-orbital localization for optimal π–π stacking with TYR38 (Figure 10). PCA/DCCM validated restricted conformational flexibility (Figure 11). ASM confirmed GLN47 and ARG136 as irreplaceable—mutations increased ΔΔG_bind_. Integrated computational analyses collectively elucidated the structural basis of compound **11**’s potent DHODH binding affinity.

Despite the comprehensive nature of this research, this study acknowledged some limitations: (1) restricted viral strain coverage (SARS-CoV-2, influenza), excluding others such as Zika viruses; (2) absence of in vivo pharmacodynamics, and pharmacokinetic and toxicity data. Future work should focus on addressing these limitations. Ultimately, we integrated a comprehensive workflow encompassing screening, computational design, synthesis, and activity evaluation, leading to the discovery of compound **11**—a structurally novel DHOD inhibitor with broad-spectrum antiviral activity. This work signified a notable advancement in the discovery strategy for DHODH inhibitors.

## 4. Materials and Methods

### 4.1. Virtual Screening

Over 1.6 million commercially accessible small molecules (ChemDiv and TargetMol databases) were prepared utilizing the LigPrep module within Schrödinger Maestro. This processing assigned standard protonation states and optimized molecular geometries via energy minimization employing the OPLS3e force field. In parallel, the crystal structure of DHODH and teriflunomide (PDB ID: 1D3H) was refined and energy-minimized using Maestro’s Protein Preparation Wizard. The prepared compound library underwent virtual screening against the optimized DHODH structure using the Glide docking module. A binding site encompassing a 20 Å cubic region centered on the co-crystallized ligand teriflunomide (**17**) was defined. Validation of the docking protocol was achieved through successful redocking of teriflunomide (**17**), yielding an RMSD of less than 1.5 Å relative to its crystallographically determined pose. Primary screening was performed using a sequential approach involving High Throughput Virtual Screening (HTVS) followed by Standard Precision (SP) docking modes. Compounds exhibiting favorable SP docking scores were subsequently evaluated using the more stringent Extra Precision (XP) methodology to improve both pose accuracy and scoring correlation for the highest-ranking ligands.

Final compound selection incorporated considerations beyond docking scores, encompassing critical physicochemical properties and detailed characterization of ligand–protein interactions within the binding pocket, including hydrogen bonding, hydrophobic contacts, and other key binding motifs. It is imperative to note that these computationally prioritized hits necessitate subsequent experimental validation, such as through in vitro assays, to confirm their activity.

### 4.2. Prime/MM–GBSA Simulation

The binding free energy (ΔG_bind_) of each ligand was computed on the basis of the MM-GBSA method [19], defined by the following equation:

ΔG_bind_ = ΔE_MM_ + ΔG_solv_ + ΔG_SA_ (1)

The term ΔE_MM_ denoted the change in molecular mechanics energy, calculated as the difference between the potential energy of the energy-minimized complex and the sum of the potential energies of the isolated, energy-minimized protein and ligand. ΔG_solv_ signified the change in solvation free energy upon complex formation, which was computed using the Generalized Born (GB) implicit solvation model. ΔG_SA_ accounts for the nonpolar solvation energy contribution, computed based on the solvent-accessible surface area (SASA) difference between the bound complex and the separated constituent systems.

### 4.3. Lipinski’s Rule and ADMET Prediction

ADMET profiling represented a central selection criterion in contemporary drug discovery workflows. Key pharmacokinetic parameters—including blood–brain barrier (BBB) permeation, gastrointestinal (GI) absorption efficiency, aqueous solubility at physiological pH, hepatotoxicity potential, and plasma protein binding (PPB) affinity—were rigorously assessed using Discovery Studio 3.0 [14,20]. Compound toxicity profiles were subsequently predicted employing the TOPKAT module. Drug-likeness was comprehensively evaluated against established criteria encompassing Lipinski’s Rule of Five and Veber’s bioavailability parameters within the integrated computational platform.

### 4.4. Materials

Compounds **1**–**10** were acquired from TargetMol Chemicals Inc. (Boston, MA, USA), while compounds **11**–**16** were synthesized in-house. MDCK cells (ATCC) and Vero-ACE2-TMPRSS2 cells (BEI Resources) were maintained in Dulbecco’s Modified Eagle Medium (DMEM) supplemented with 10% (*v/v*) fetal bovine serum (FBS) at 37 °C with 5% CO_2_. DHODH protein was obtained by Shanghai Bohu Biological Technology Co.; Ltd. (Shanghai, China).

### 4.5. Cytotoxicity

The cytotoxic effects of the test compounds on MDCK and Vero cell lines were evaluated utilizing the 3-(4,5-dimethylthiazol-2-yl)-2,5-diphenyltetrazolium bromide (MTT) assay [21]. In brief, cells were seeded in 96-well plates and maintained in complete growth medium. After an overnight incubation period, this medium was aspirated and replaced with serum-free DMEM containing the compounds at predetermined concentrations. After 48 h of treatment, MTT dissolved in PBS was introduced into each well to achieve a final concentration of 0.5 mg/mL. Subsequent incubation at 37 °C for 2 h facilitated formazan crystal formation. The medium was then aspirated, and DMSO was added to solubilize the formazan crystals. Absorbance measurements at 540 nm were conducted using a CLARIOstar microplate reader.

### 4.6. Viral Titre Determination by Plaque Assay

Anti-influenza activity of compounds was tested on MDCK cells using plaque reduction assay. A 10-fold serial dilution series was initially prepared for each virus prior to titration. Preseeded MDCK cells were incubated with serially diluted viral inocula for one hour, subsequently washed with PBS, and overlaid with a 1:1 mixture of 2% agarose, serum-free minimum essential medium (MEM), and 1 µg/mL L-(tosylamido-2-phenyl) ethyl chloromethyl ketone (TPCK)-treated trypsin, with the exception of the A/PR/8/34(H1N1) influenza strain, where TPCK-trypsin was excluded. Following incubation at 37 °C for 48 h, the agarose overlay was removed and cells were fixed and stained with 0.1% (*w/v*) Coomassie Brilliant Blue in 30% methanol and 5% glacial acetic acid. Plaques were enumerated, and viral titers were calculated and expressed as plaque-forming units per milliliter (PFU/mL).

The antiviral activity against SARS-CoV-2 was evaluated in Vero-ACE2-TMPRSS2 cells, which were engineered to stably express human angiotensin-converting enzyme 2 (ACE2) and transmembrane serine protease 2 (TMPRSS2) to enhance viral susceptibility. After plating in 24-well plates, cells underwent two quick washes with PBS prior to the infection step. A viral stock of the SARS-CoV-2 strain was diluted in plain DMEM to achieve approximately 40–50 plaque-forming units (PFUs) per well. The cells were inoculated with the diluted virus and incubated at 37 °C for 1.5 h. Test compounds were serially diluted four-fold in plain DMEM. Following viral adsorption, a mixture consisting of 2% agarose and the diluted compound solution was prepared at a 1:1 ratio and overlaid onto the infected cell monolayer. The plates were subsequently inverted and incubated at 37 °C under 5% CO_2_ for 24–36 h to facilitate plaque formation.

Following the formation of plaques under both experimental conditions, cell monolayers were subjected to fixation with 4% paraformaldehyde for a period of two hours at room temperature and were subsequently treated with a 1% crystal violet solution for staining, according to a standard protocol. After rinsing gently under running tap water, viral plaques were enumerated manually. The percentage inhibition of viral infection was calculated relative to a PBS-treated control group using the following equation: % Inhibition = [1 − (Number of plaques in compound-treated well/Number of plaques in PBS control well)] × 100%. The half-maximal inhibitory concentration (IC_50_) was determined by nonlinear regression analysis using GraphPad Prism version 9.5.

### 4.7. Surface Plasmon Resonance Experiment

SPR binding affinities were determined using a Reichert 2SPR instrument (Reichert Technologies, Amherst, NY, USA). Carboxymethyl dextran hydrogel sensor chips were amine-coupled via standard EDC/NHS chemistry. DHODH was immobilized in 10 mM sodium acetate (pH 4.5) to achieve a response unit (RU) value of 4000 in flow channel 1. Remaining activated groups were quenched with 1 M ethanolamine-HCl (pH 8.5). Equilibrium dissociation constants (*K*_D_) were derived from concentration–response curves generated by injecting compound dilutions in HBS-EP+ buffer at 30 μL/min. Original data were processed using TraceDrawer (version 1.8) software with steady-state affinity models fitted by nonlinear regression analysis.

### 4.8. MD Simulations

The complexes underwent 100 ns MD simulations using the Desmond software (version 3.8, Schrödinger Suite 2016-1, Schrödinger, LLC, New York, NY, USA), employing the OPLS_2005 force field. Simple Point Charge (SPC) water molecules and neutralizing counterions were added to each system [22,23,24,25]. Energy minimization was performed with a convergence threshold applied to the gradient and a maximum limit of 5000 iterations. Production simulations were conducted under isothermal-isobaric (NPT) ensemble conditions at 300 K and 1 atm (1.01325 bar). The trajectory data were analyzed using Desmond’s Simulation Quality Analysis, Simulation Event Analysis, and Simulation Interaction Diagram tools to evaluate simulation convergence and characterize protein–ligand binding modes.

### 4.9. ASM

ASM employed single-site alanine substitutions to delineate the functional contributions of specific amino acid residues within binding interfaces [26,27,28,29]. The energetic perturbation to binding affinity, quantified by the difference in binding free energy between the mutant and wild-type complex (ΔΔG_bind_ = ΔG_bind,mutant_ − ΔG_bind,wild type_), serves as a critical experimental benchmark for verifying predictions derived from computational free energy decomposition studies. A positive ΔΔG_bind_ value signifies increased complex destabilization upon mutation, thereby thermodynamically implicating the substituted residue as functionally critical for ligand binding.

### 4.10. QM/MM Calculations

QM/MM calculations were carried out in Schrödinger’s QSite (2023-1). The QM region, encompassing the reactive site, was computed with DFT/B3LYP-D3/6-31G*, while the MM environment (protein, solvent, ions) was modeled with the OPLS4 force field. The QM/MM interface was managed using link atoms with hydrogen caps. Structural stability for the outer MM region was ensured by applying harmonic restraints (0.5 kcal·mol^−1^·Å^−2^) to heavy atoms beyond 15 Å from the QM boundary. Potential energy surfaces were mapped via constrained optimizations along predefined reaction coordinates to locate approximate transition state geometries. Initial TS structures underwent refinement using the quadratic synchronous transit algorithm, with subsequent vibrational frequency analysis verifying the existence of a single imaginary mode. Gibbs free energy barriers were determined through umbrella sampling simulations (50 windows, 100 ps/window), with potentials of mean force (PMFs) derived via the weighted histogram analysis method (WHAM). Natural Bond Orbital (NBO) theory was applied to quantify charge distributions, bond orders, and orbital hybridization effects within the QM subsystem.

### 4.11. DCCM Analysis and PCA

To assess correlated residue motions within the MD trajectory ensemble, a DCCM was calculated from the Cα backbone atomic trajectories using the Bio3D package. Limiting analysis to Cα atoms minimized statistical noise and side-chain conformational artifacts. For residue pairs *i* and *j*, the normalized covariance coefficient *C_ij_* quantifies correlated displacements:

*C_ij_* = Δr_i_ ∗ Δr_j_/(Δr_i_^2^ ∗ Δr_j_^2^)^1/2^ (2)

The correlation coefficient *Cᵢⱼ* is computed from the instantaneous displacement vectors (Δr_i_ and Δr_j_) of residues *i* and *j* from their respective mean positions. Its value spans from −1.0, indicating perfectly anti-correlated motion, to +1.0, indicating perfectly correlated motion, thereby directly quantifying the degree of concerted dynamics between residue pairs.

PCA was additionally conducted via the Bio3D package implemented in the R statistical environment to characterize dominant collective motions and identify essential conformational subspaces within the MD trajectory ensemble [30,31].

### 4.12. Chemistry

The melting points were measured in open capillaries using an X-4 digital melting point apparatus and were reported without correction. We performed high-resolution mass spectrometric analyses on an Agilent Technologies 6530 Q-TOF instrument (Santa Clara, CA, USA) configured with a dual electrospray ionization (ESI) source. ^1^H NMR or ^13^C NMR spectra were recorded at 600 MHz or 151 MHz on a Bruker spectrometer (Fällanden, Switzerland) using tetramethylsilane (TMS) as the internal standard and dimethyl sulfoxide-d_6_ (DMSO-*d_6_*) or deuterated chloroform (CDCl_3_) as solvents. Chemical shifts (*δ*) were reported in parts per million (ppm) and were referenced relative to residual solvent signals. Coupling constants (*J*) were expressed in Hertz (Hz). The corresponding NMR spectra are available within the Supplementary Information.

All commercial solvents and reagents were acquired from established suppliers and employed in reactions without additional purification.

#### 4.12.1. Preparation of Compounds **20a**–**20c**

A mixture of 5-bromopyridin-2-amine derivatives (**19a** or **19b** or **19c**, 22.73 mmol) in anhydrous ethanol (30 mL) was prepared in a 250 mL flask equipped with stirrer. Separately, ethyl α-chloroacetoacetate (56.81 mmol) was dissolved in anhydrous ethanol (20 mL), and the resulting solution was added dropwise to the stirred pyridine mixture at room temperature over 15 min. Subsequently, additional anhydrous ethanol (50 mL) was introduced into the reaction vessel. The flask was fitted with a reflux condenser, and the reaction mixture was heated under vigorous reflux (78 °C) for 63 h. Upon completion, the mixture was cooled to room temperature, and insoluble impurities were removed by vacuum filtration. The filtrate was concentrated under reduced pressure to afford a crude solid. This residue was suspended in acetone (15 mL) and stirred vigorously for 30 min at room temperature. The suspension was subjected to vacuum filtration, and the collected solid was washed sequentially with acetone (2 × 10 mL) until the eluent appeared colorless. The washed solid was dried under high vacuum at 50 °C for 12 h to yield ethyl 6-bromo-2-methylimidazo[1,2-*a*]pyridine-3-carboxylate derivatives (**20a**–**20c**).

Ethyl 6-bromo-2-methylimidazo[1,2-*a*]pyridine-3-carboxylate (**20a**), yellow powder, yield 54.8%. ^1^H NMR (600 MHz, DMSO-*d*_6_) δ 9.40 (s, 1H), 8.04 (s, 1H), 4.42 (q, *J* = 7.1 Hz, 2H), 2.68 (d, *J* = 2.1 Hz, 3H), 1.39 (t, *J* = 7.1 Hz, 3H).

Ethyl 6-bromo-2,7-dimethylimidazo[1,2-*a*]pyridine-3-carboxylate (**20b**), yellow powder, yield 37.5%. ^1^H NMR (600 MHz, DMSO-*d*_6_) δ 9.45 (s, 1H), 8.00 (s, 1H), 4.43 (t, *J* = 7.0 Hz, 2H), 2.71 (s, 3H), 2.57 (s, 3H), 1.39 (t, *J* = 7.1 Hz, 3H).

Ethyl 6-bromo-2-methylimidazo[1,2-*a*]pyrimidine-3-carboxylate (**20c**), yellow powder, yield 24.6%. ^1^H NMR (600 MHz, DMSO-*d*_6_) δ 9.53 (s, 1H), 8.30 (s, 1H), 4.39 (q, *J* = 7.1 Hz, 2H), 2.63 (s, 3H), 1.37 (t, *J* = 7.1 Hz, 3H).

#### 4.12.2. Preparation of Compounds **14**–**16**

A mixture of 6-bromo-2-methylimidazo[1,2-*a*]pyridine-3-carboxylate derivatives (**20a**–**20c**, 0.0020 mol), the appropriate arylboronic acid (0.0020 mol), tetrakis(triphenylphosphine)palladium(0) (Pd(PPh_3_)_4_, 0.23 g, 0.00020 mol), and cesium carbonate (Cs_2_CO_3_, 1.95 g, 0.0060 mol) was added. 1,4-Dioxane (30 mL) and water (2 mL) were added to dissolve the solids. The reaction mixture was degassed with argon for 10–15 min and then heated to 120 °C with vigorous stirring under an argon atmosphere. The progress of the reaction was monitored by thin-layer chromatography (TLC). Upon completion, the reaction mixture was cooled to room temperature. The mixture was then diluted with ethyl acetate (or dichloromethane, ~30 mL) and filtered through a pad of Celite, washing thoroughly with additional solvent. The filtrate was concentrated under reduced pressure. The crude residue was purified by flash column chromatography on silica gel to afford **14**–**16**.

Ethyl 6-([1,1′-biphenyl]-4-yl)-2-methylimidazo[1,2-*a*]pyridine-3-carboxylate (**14**), white powder, yield 25.6%. ^1^H NMR (600 MHz, Chloroform-*d*) δ 9.63 (s, 1H), 7.70 (dd, *J* = 5.1, 1.6 Hz, 6H), 7.66–7.62 (m, 2H), 7.50–7.45 (m, 2H), 7.40–7.35 (m, 1H), 4.46 (q, *J* = 7.1 Hz, 2H), 2.75 (s, 3H), 1.46 (t, *J* = 7.1 Hz, 3H). ^13^C NMR (151 MHz, Chloroform-*d*) δ 161.59, 153.26, 146.20, 140.93, 135.99, 128.90, 127.83, 127.76, 127.60, 127.42, 127.07, 125.42, 116.53, 112.96, 60.37, 16.83, 14.52. ESI-MS *m/z*: calculated C_23_H_20_N_2_O_2_ ([M+H]^+^): 357.16, found: 357.16. m.p.: 188–190 °C.

Ethyl 6-([1,1′-biphenyl]-4-yl)-2,7-dimethylimidazo[1,2-*a*]pyridine-3-carboxylate (**15**), white powder, yield 16.9%. ^1^H NMR (600 MHz, Chloroform-*d*) δ 9.21 (s, 1H), 7.70–7.63 (m, 4H), 7.51–7.45 (m, 3H), 7.45–7.35 (m, 3H), 4.40 (q, *J* = 7.1 Hz, 2H), 2.73 (s, 3H), 2.38 (s, 3H), 1.42 (t, *J* = 7.1 Hz, 3H). ^13^C NMR (151 MHz, DMSO-*d*_6_) δ161.11, 152.73, 146.43, 140.17, 139.98, 136.34, 130.51, 129.52, 128.19, 127.33, 127.22, 126.36, 116.30, 111.90, 60.47, 20.78, 16.87, 14.74. ESI-MS *m/z*: calculated C_24_H_22_N_2_O_2_ ([M+H]^+^): 371.17, found: 371.17. m.p.: 168–170 °C.

Ethyl 6-([1,1′-biphenyl]-4-yl)-2-methylimidazo[1,2-*a*]pyrimidine-3-carboxylate (**16**), white powder, yield 23.5%. ^1^H NMR (600 MHz, DMSO-*d*_6_) δ 9.68 (d, *J* = 2.6 Hz, 1H), 9.14 (d, *J* = 2.6 Hz, 1H), 7.92–7.82 (m, 4H), 7.79–7.74 (m, 2H), 7.52–7.45 (m, 2H), 7.40–7.37 (m, 1H), 4.42 (q, *J* = 7.1 Hz, 2H), 2.69 (s, 3H), 1.40 (t, *J* = 7.1 Hz, 3H). ^13^C NMR (151 MHz, DMSO-*d*_6_) δ 160.84, 152.19, 139.70, 132.73, 129.54, 128.32, 128.11, 127.95, 127.18, 123.20, 111.30, 60.99, 16.84, 14.71. ESI-MS *m/z*: calculated C_22_H_19_N_3_O_2_ ([M+H]^+^): 358.14, found: 358.14. m.p.: 196–197 °C.

#### 4.12.3. Preparation of Compounds **11**–**13**

A 250 mL flask was charged with ethyl 6-([1,1′-biphenyl]-4-yl)-2-methylimidazo[1,2-*a*]pyridine-3-carboxylate derivatives (**14**–**16**, 10.15 mmol), distilled water (30 mL), and ethanol (30 mL). A separate solution of sodium hydroxide (50.75 mmol) in distilled water (20 mL) was prepared and added dropwise to the stirred reaction mixture at room temperature. The resulting mixture was then heated to 60 °C with vigorous stirring. After 6 h, the mixture was cooled to room temperature. The ethanol was removed under reduced pressure, and the residual aqueous suspension was subjected to vacuum filtration to eliminate insoluble impurities. The filtrate was acidified to pH 4–5 with concentrated HCl, inducing precipitation of a solid product. The suspension was filtered under vacuum, and the collected filter cake was washed sequentially with distilled water (2 × 15 mL) to remove residual salts. The solid was dried under high vacuum (0.5 mbar) at 50 °C for 12 h to afford **11**–**13**.

6-([1,1′-Biphenyl]-4-yl)-2,7-dimethylimidazo[1,2-*a*]pyridine-3-carboxylic acid (**11**), white powder, yield 25.5%. ^1^H NMR (600 MHz, DMSO-*d*_6_) δ 9.28 (s, 1H), 8.00 (s, 1H), 7.85 (d, *J* = 8.2 Hz, 2H), 7.76 (d, *J* = 7.0 Hz, 2H), 7.59 (d, *J* = 8.2 Hz, 2H), 7.53–7.50 (m, 2H), 7.45–7.39 (m, 1H), 2.76 (s, 3H), 2.50 (s, 3H). ^13^C NMR (151 MHz, DMSO-*d*_6_) δ 161.59, 145.12, 140.83, 140.02, 139.78, 134.84, 132.57, 130.48, 129.53, 128.34, 127.59, 127.55, 127.26, 113.73, 112.26, 20.99, 13.12. ESI-MS *m/z*: calculated C_22_H_18_N_2_O_2_ ([M+H]^+^): 343.14, found: 343.14. m.p.: 220–221 °C.

6-([1,1′-Biphenyl]-4-yl)-2-methylimidazo[1,2-*a*]pyridine-3-carboxylic acid (**12**), white powder, yield 33.6%. ^1^H NMR (600 MHz, DMSO-*d*_6_) δ 9.68 (s, 1H), 8.21 (d, *J* = 9.1 Hz, 1H), 7.98 (d, *J* = 9.2 Hz, 1H), 7.90–7.84 (m, 3H), 7.76 (d, *J* = 7.8 Hz, 2H), 7.52 (s, 2H), 7.42 (s, 1H), 2.72 (s, 3H). ESI-MS *m/z*: calculated C_21_H_16_N_2_O_2_ ([M+H]^+^): 329.13, found: 329.13. m.p.: 217–219 °C.

6-([1,1′-Biphenyl]-4-yl)-2-methylimidazo [1,2-*a*]pyrimidine-3-carboxylic acid (**13**), white powder, yield 21.2%.^1^H NMR (600 MHz, DMSO-*d*_6_) δ 9.75 (d, *J* = 2.6 Hz, 1H), 9.15 (d, *J* = 2.6 Hz, 1H), 8.72 (s, 1H), 7.88 (s, 3H), 7.75 (s, 3H), 7.51 (d, *J* = 7.9 Hz, 2H), 2.69 (s, 3H). ESI-HRMS *m/z*: calculated C_20_H_15_N_3_O_2_ ([M+H]^+^): 330.1298, found: 330.1235. m.p.: 242–243 °C.

## 5. Conclusions

Taking all the data together, we concluded that compound **11** was a highly effective DHODH inhibitor with a novel structural framework, offering a promising strategy for broad-spectrum antiviral therapy against RNA viruses by depleting pyrimidine pools essential for viral replication.

## Figures and Tables

**Table 1:** Summary of in vitro screening results for the test compounds.

No.	Structure	Score (kcal/mol)	MM/GBSA (kcal/mol)	A/PR/8/34(H1N1)		
				CC_50_ (μM)	IC_50_ (μM)	SI
**1**	** 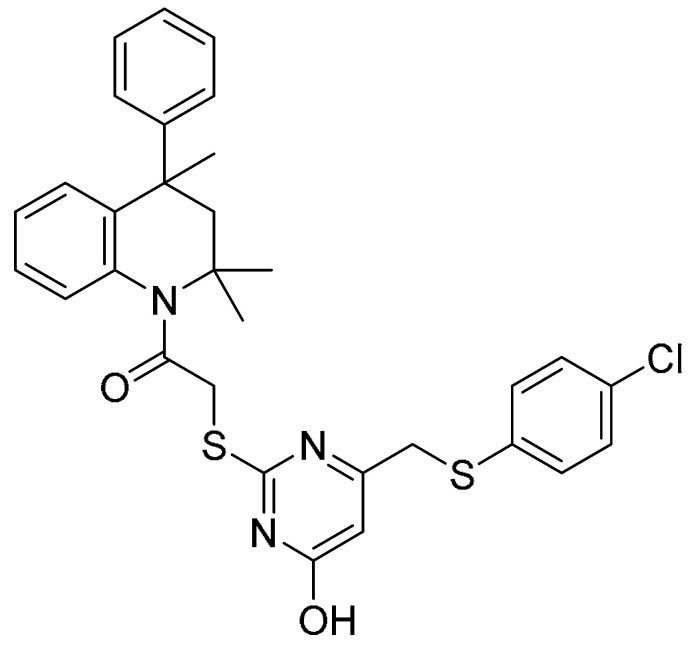 **	−12.294	−53.78	34.52 ± 1.58	36.83 ± 2.36	0.9
**2**	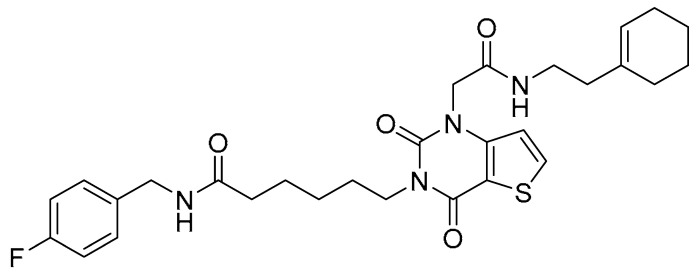	−12.181	−74.80	>200	>200	-
**3**	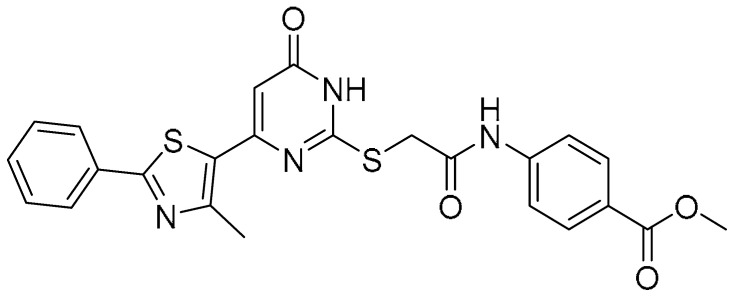	−11.570	−61.61	>200	>200	-
**4**	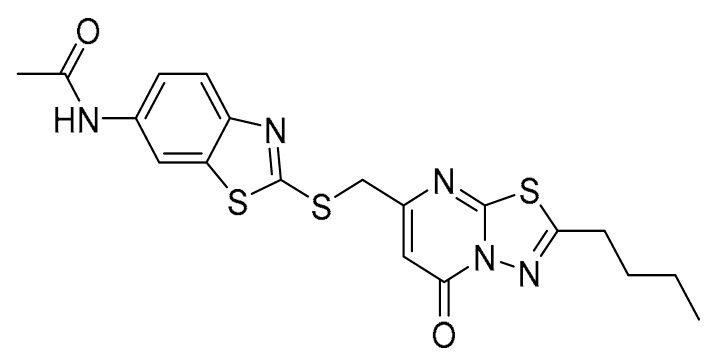	−9.904	−83.20	>200	66.15 ± 6.29	>3.0
**5**	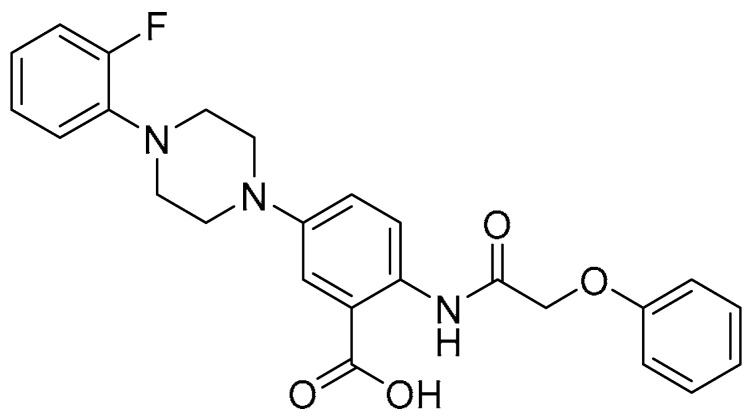	−10.351	−67.08	>200	>200	-
**6**	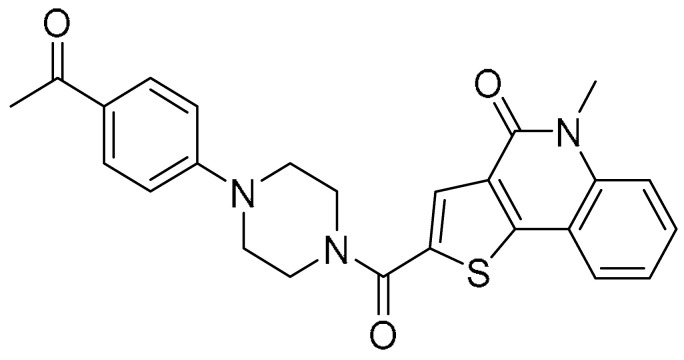	−12.113	−82.85	>200	4.85 ± 0.58	>41.2
**7**	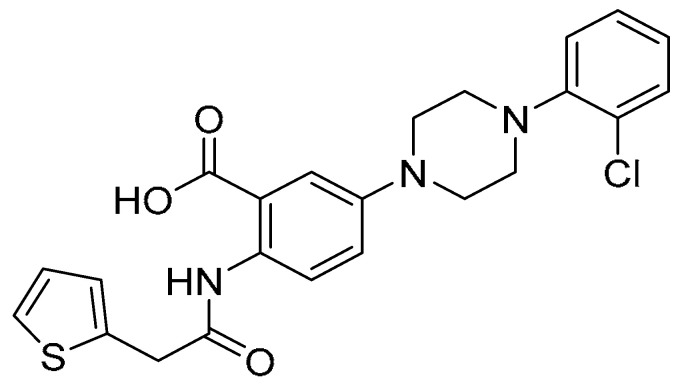	−11.761	−66.23	>200	17.20 ± 1.19	>11.6
**8**	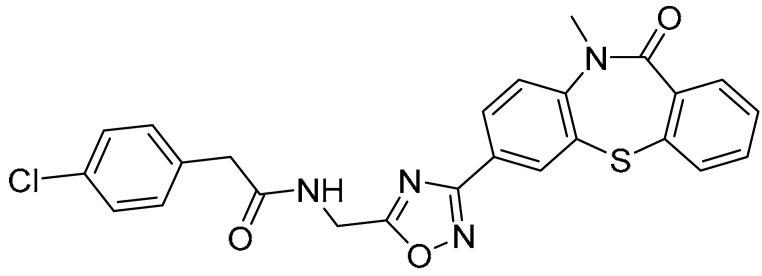	−11.705	−80.56	>200	13.38 ± 2.45	>14.9
**9**	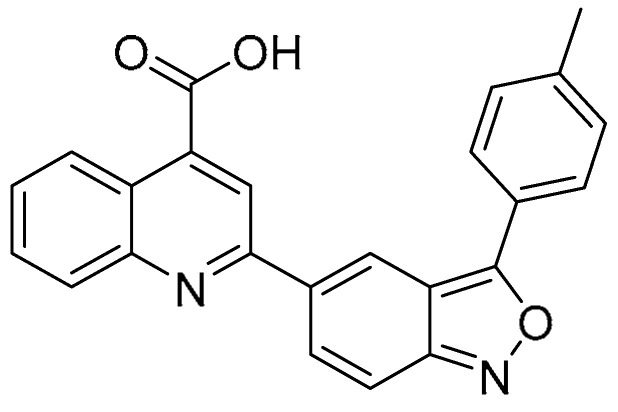	−13.497	−73.06	>200	7.35 ± 1.65	>21.2
**10**	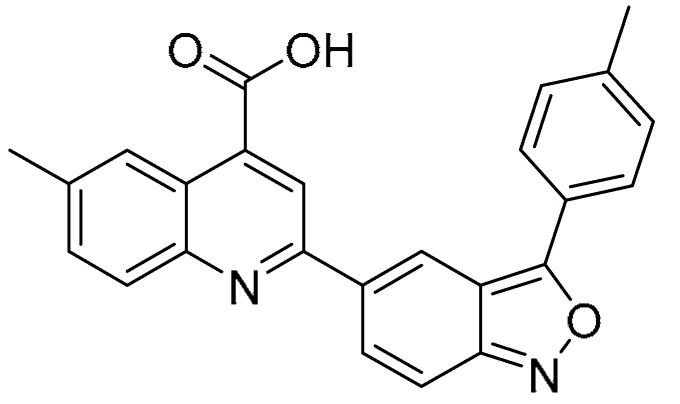	−13.077	−70.25	>200	1.75 ± 0.28	>114.3
**17**	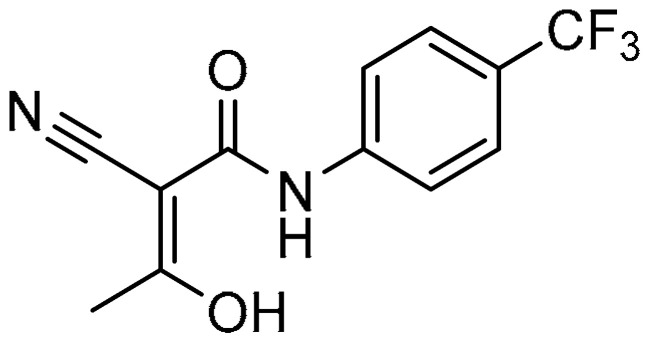	−9.100	−44.01	180.90 ± 16.45	35.02 ± 3.33	5.2

**Table 2:** Bioactivity evaluation results of the active compounds.

No.	Structure	A/PR/8/34(H1N1)			SARS-CoV-2		
		CC_50_ (μM)	IC_50_ (μM)	SI	CC_50_ (μM)	IC_50_ (μM)	SI
**11**	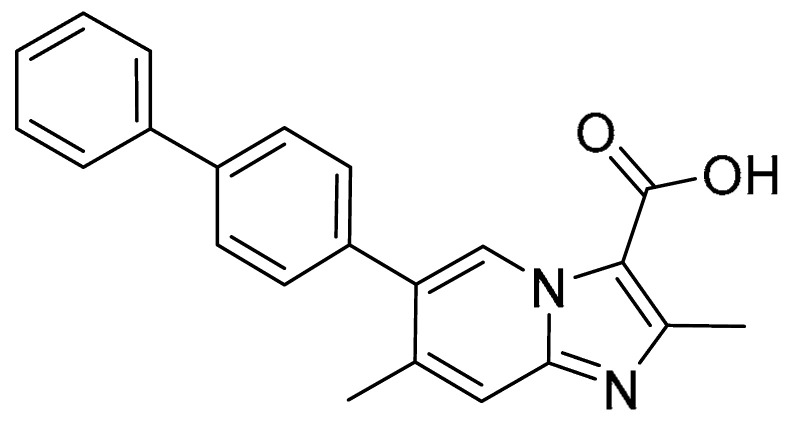	>200	0.85 ± 0.05	>235.3	>200	3.60 ± 0.67	>55.6
**12**	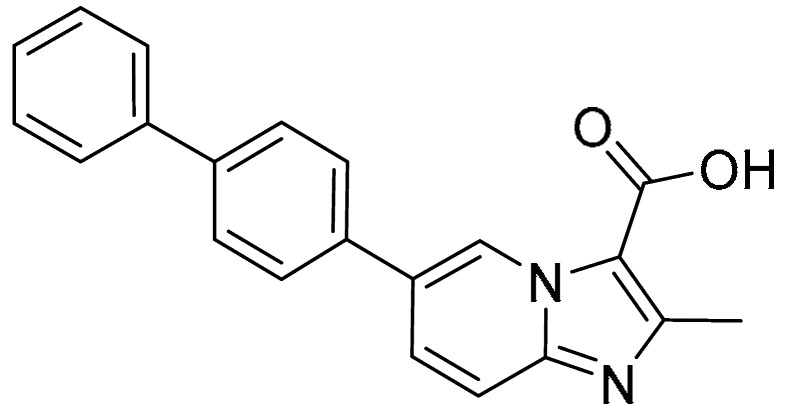	>200	100 ± 1.13	>2.0	>200	>200	-
**13**	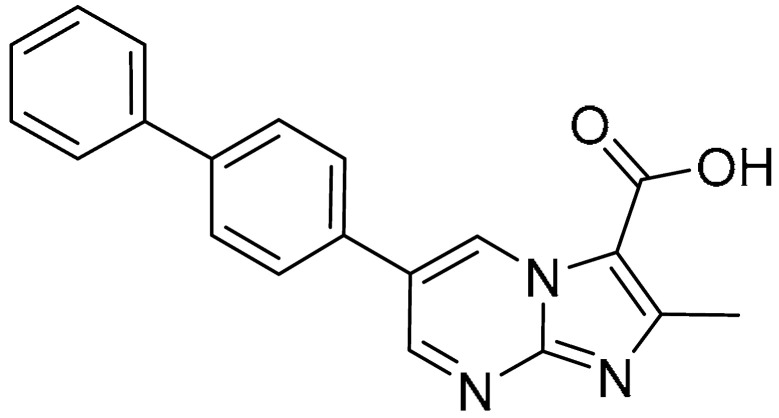	>200	1.08 ± 0.26	>185.2	>200	104.84 ± 5.19	>1.9
**14**	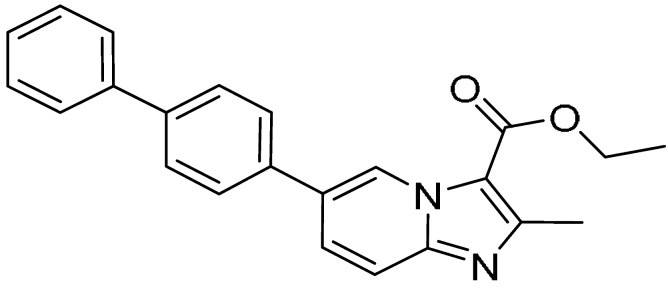	>200	>200	-	>200	>200	-
**15**	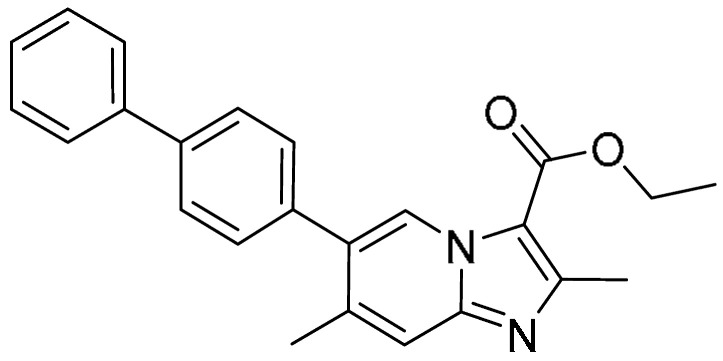	>200	>200	-	>200	>200	-
**16**	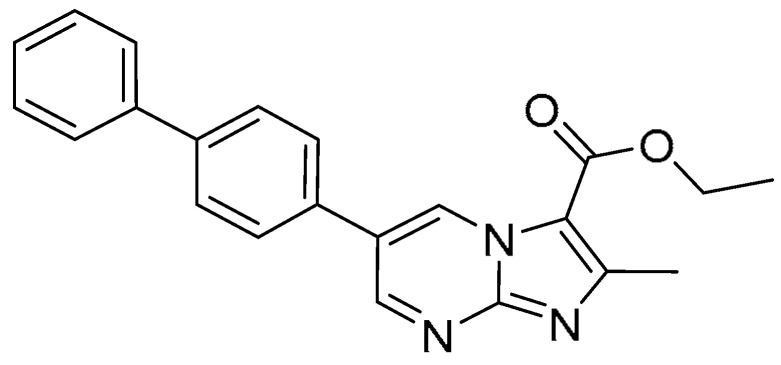	>200	>200	-	>200	>200	-
**17**	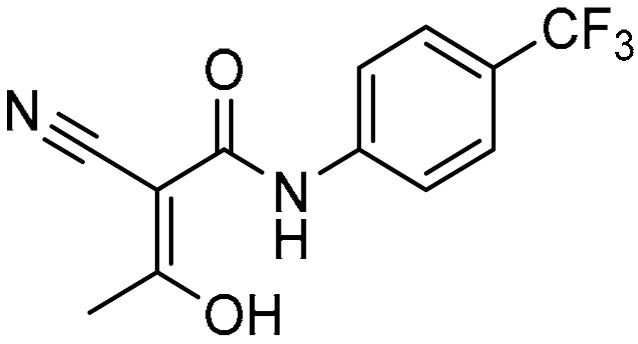	180.90 ± 16.45	35.02 ± 3.33	5.2	>200	26.06 ± 4.32	>7.7
Baloxavir marboxil	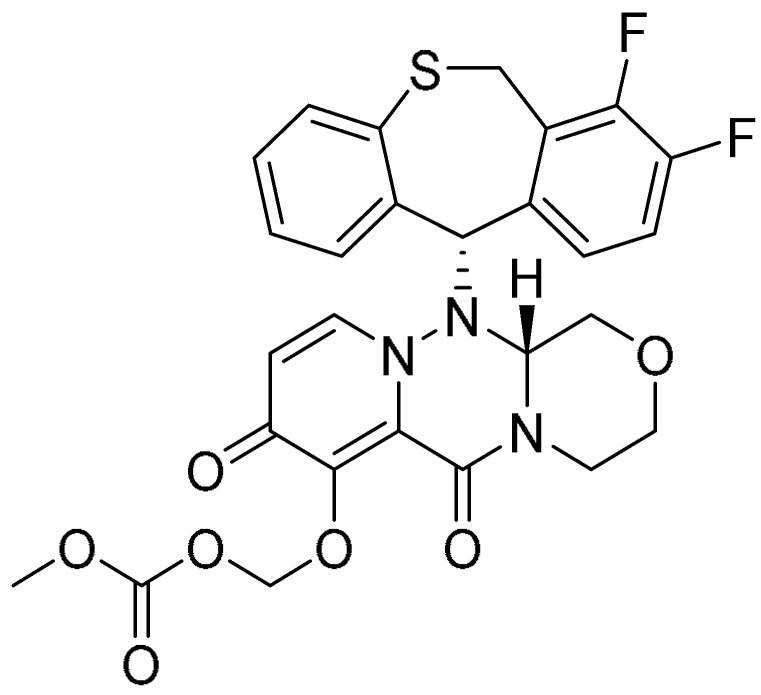	-	9.67 ± 1.12 nM	-	-	-	-
Remdesivir	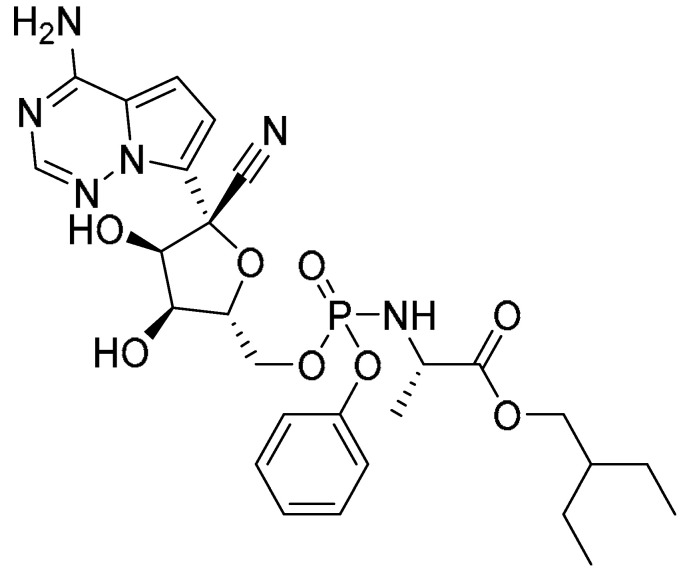	-	-	-	-	4.95 ± 0.78	-

**Table 3:** The kinetic parameters and binding affinities of compounds with DHODH.

No.	K_a_ (M^−1^S^−1^)	K_dis_ (S^−1^)	*K*_D_ (M)
**11**	4.83 × 10^6^	2.93 × 10^1^	6.06 × 10^6^
**17**	3.36 × 10^9^	8.04 × 10^4^	2.39 × 10^5^
**18**	1.31 × 10^6^	8.02 × 10^0^	6.13 × 10^6^

K_a_, association constant; K_dis_, dissociation constant.

## Data Availability

Data is contained within the article and Supplementary Materials.

## Supplementary Materials

The following supporting information can be downloaded at https://www.mdpi.com/article/10.3390/ph18091416/s1, Table S1. The ADMET prediction results for the candidate compounds (**1**–**10**), **17**, and **18**; Table S2. Toxicity predictions of the candidate compounds (**1**–**10**), **17**, and **18**; Table S3. The results of Lipinski’s rule calculation for the candidate compounds (**1**–**10**), **17**, and **18**; Table S4. Further spectral details of compounds **11**–**16**; Figure S1. Alignment of redocked (cyan) and crystallographic (pink) ligand in DHODH active site validating docking precision; Figure S2. RMSD of candidate compounds (**1**–**10**) and **17** complexes over 20 ns.

## Abbreviations

The following abbreviations are used in this manuscript:

DHODH	Dihydroorotate dehydrogenase
hDHODH	Human DHODH
DENV	Dengue viruses
ZIKV	Zika viruses
MD	Molecular dynamics
QM/MM	Quantum mechanics/molecular mechanics
MM/GBSA	Molecular Mechanics/Generalized Born Surface Area
SPR	Surface Plasmon Resonance
RMSD	Root-mean-square deviation
RMSF	Root-mean-square fluctuation
rGyr	Radius of gyration
MolSA	Molecular surface area
SASA	Solvent-accessible surface area
PSA	Polar surface area
HOMO	Highest occupied molecular orbital
LUMO	Lowest unoccupied molecular orbital
PCA	Principal component analysis
DCCM	Dynamic cross-correlation matrices
HTVS	High Throughput Virtual Screening
SP	Standard Precision
XP	Extra Precision
GB	Generalized Born
BBB	Blood–brain barrier
GI	Gastrointestinal
PPB	Plasma protein binding
DMEM	Dulbecco’s modified Eagle’s medium
FBS	Fetal bovine serum
MTT	3-(4,5-Dimethylthiazol-2-yl)-2,5-diphenyltetrazolium bromide
MEM	Minimum essential medium
RU	Response unit
SPC	Simple Point Charge
ASM	Alanine scanning mutagenesis
PMFs	Potentials of mean force
WHAM	Weighted histogram analysis method
NBO	Natural Bond Orbital
TLC	Thin-layer chromatography
*K*_D_	Dissociation constants
MDCK	Madin–Darby Canine Kidney
SAR	Structure–activity relationship
