# Supplementary material for: Comprehensive Identification and Mechanistic Evaluation of Novel DHODH Inhibitors as Potent Broad-Spectrum Antiviral Agents
Source: Pharmaceuticals (Basel). 2025 Sep 20;18(9):1416. doi: 10.3390/ph18091416 (PMC12472551; doi:10.3390/ph18091416)
Supplement: Supplementary file 1 [file pharmaceuticals-18-01416-s001.zip › pharmaceuticals-3836891-supplementary.pdf]

Supplementary Material

Comprehensive Identification and Mechanistic Evaluation of Novel DHODH Inhibitors as Potent Broad-Spectrum Antiviral Agents

Chao Zhang [1], Shiyang Sun [1], Huiru Xie [1], Yongzhao Ding [1,2], Chun Hu [2], Jialin Guo [1,*] and Junhai Xiao [1,*]

[1] State Key Laboratory of National Security Specially Needed Medicines, Beijing 100039, China; zhangchaoylh@126.com (C.Z.); noah97sun@163.com (S.S.); xiehuiru0808@163.com (H.X.); 13308109105@163.com (Y.D.)

[2] Key Laboratory of Structure-Based Drug Design Discovery, Ministry of Education, School of Pharmaceutical Engineering, Shenyang Pharmaceutical University, Shenyang 110016, China; chunhu@syphu.edu.cn

* Correspondence: shandongguojialin@163.com (J.G.); xiaojunhai@139.com (J.X.)

Contents

Figure S1. Alignment of redocked (cyan) and crystallographic (pink) ligand in DHODH active site validating docking precision...3

Figure S2. RMSD of candidate compounds (**1-10**) and **17** complexes over 20 ns. 3

Table S1. The ADMET prediction results for the candidate compounds (**1-10**), **17** and **18**...4

Table S2. Toxicity predictions of the candidate compounds (**1-10**), **17** and **18**...4

Table S3. The results of Lipinski's rule calculation for the candidate compounds (**1-10**), **17** and **18**...5

Table S4. The further spectral details of compounds **11-16**..................................5

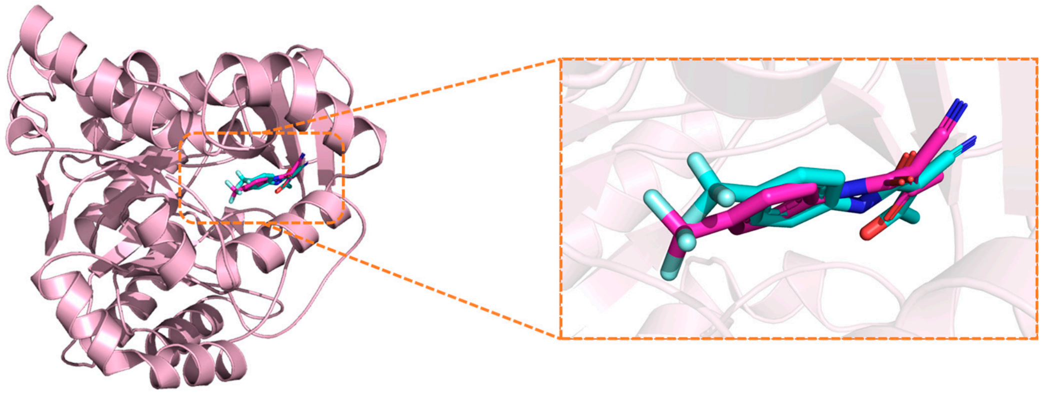

Figure S1. Alignment of redocked (cyan) and crystallographic (pink) ligand in DHODH active site validating docking precision.

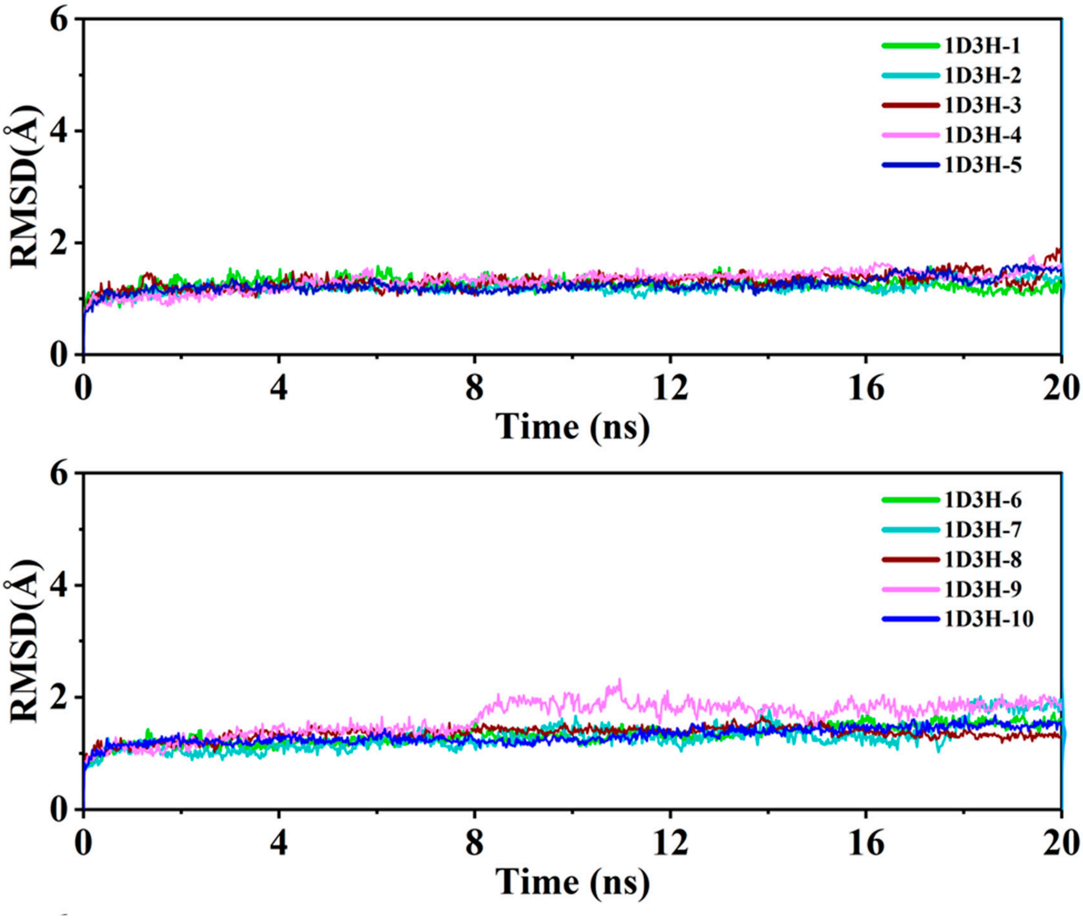

Figure S2. RMSD of candidate compounds (**1-10**) and **17** complexes over 20 ns.

Table S1. The ADMET prediction results for the candidate compounds (**1–10**), **17** and **18**.

No.	ADME Solubility Level	ADME BBB Level	ADME Absorption Level	PPB Prediction	CYP2D6 Prediction
**1**	1	4	3	true	false
**2**	2	4	1	false	false
**3**	2	4	0	false	false
**4**	2	2	0	false	false
**5**	2	2	0	true	false
**6**	2	2	0	true	false
**7**	2	1	0	true	false
**8**	2	2	0	true	false
**9**	1	4	1	true	false
**10**	1	4	1	true	false
**17**	1	2	2	true	false
**18**	1	0	0	true	true

ADME_Solubility_Level: 0 (Extremely low); 1 (No, very low, but possible); 2 (Yes, low); 3 (Yes, good); 4 (Yes, optimal); 5(No, too soluble); 6 (Warning: molecules with one or more unknown AlogP98 types).
ADME_BBB_Level: 0 (Very high penetrant); 1 (High); 2 (Medium); 3 (Low); 4 (Undefined).
ADME_Absorption_Level: 0 (Good absorption); 1 (Moderate absorption); 2 (Low absorption); 3 (Very low absorption).
EXT_PPB_Prediction: plasma protein binding ability, false: ≥ 90 %; true: ≤ 90 %.
EXT_CYP2D6_Prediction: false: non-inhibitor; true: inhibitor.

Table S2. Toxicity predictions of the candidate compounds (**1–10**), **17** and **18**.

No.	NTP carcinogenicity male Rat	NTP carcinogenicity female Rat	NTP carcinogenicity Call (Male mouse)	NTP carcinogenicity Call (Female mouse)	Ames mutagenicit y	Rat oral LD50 (in g/kg)	Skin irritation
**1**	NC	NC	NC	C	NM	0.812213	None
**2**	NC	NC	C	NC	NM	3.67291	None
**3**	NC	NC	C	NC	NM	8.32762	None
**4**	NC	NC	C	C	NM	2.22026	None
**5**	NC	NC	NC	NC	NM	4.35083	None
**6**	NC	NC	NC	NC	NM	1.14701	None
**7**	NC	NC	C	NC	NM	18.8904	None
**8**	NC	NC	NC	NC	NM	2.13583	None
**9**	C	C	NC	NC	NM	1.84156	None
**10**	C	C	NC	NC	NM	1.25448	None
17	NC	NC	C	NC	NM	1.50042	None
18	C	NC	NC	NC	NM	0.229656	None

C: Carcinogen; NC: Non-Carcinogen; NM: Non-Mutagen.

Table S3. The results of Lipinski's rule calculation for the candidate compounds (**1–10**), **17** and **18**.

No.	Alop	MW	No. HBA	No. HBD	No. rotatable bonds
**1**	7.861	576.17	6	1	7
**2**	4.647	554.68	4	2	13
**3**	3.649	492.57	7	2	8
**4**	4.18	445.58	7	1	7
**5**	4.076	449.47	6	2	7
**6**	3.537	445.53	4	0	3
**7**	4.688	455.96	5	2	6
**8**	4.183	490.96	5	1	5
**9**	5.679	380.40	4	1	3
**10**	6.166	394.42	4	1	3
**17**	2.091	270.21	3	2	3
**18**	5.803	357.38	3	1	3

MW: molecular weight; HBA: H-bond acceptors; HBD: H-bond donors.

Table S4. The further spectral details of compounds **11–16.**

**Compound 11**	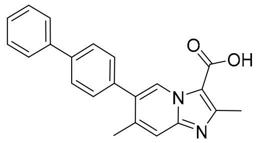
**MS**	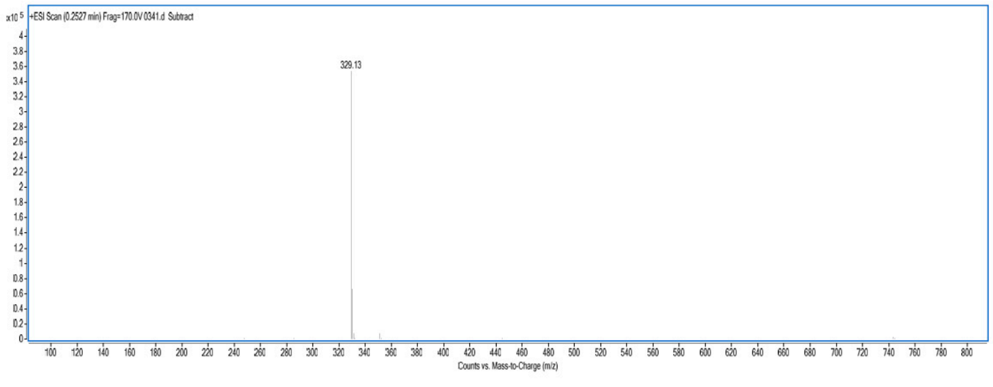

**^{1}H NMR**	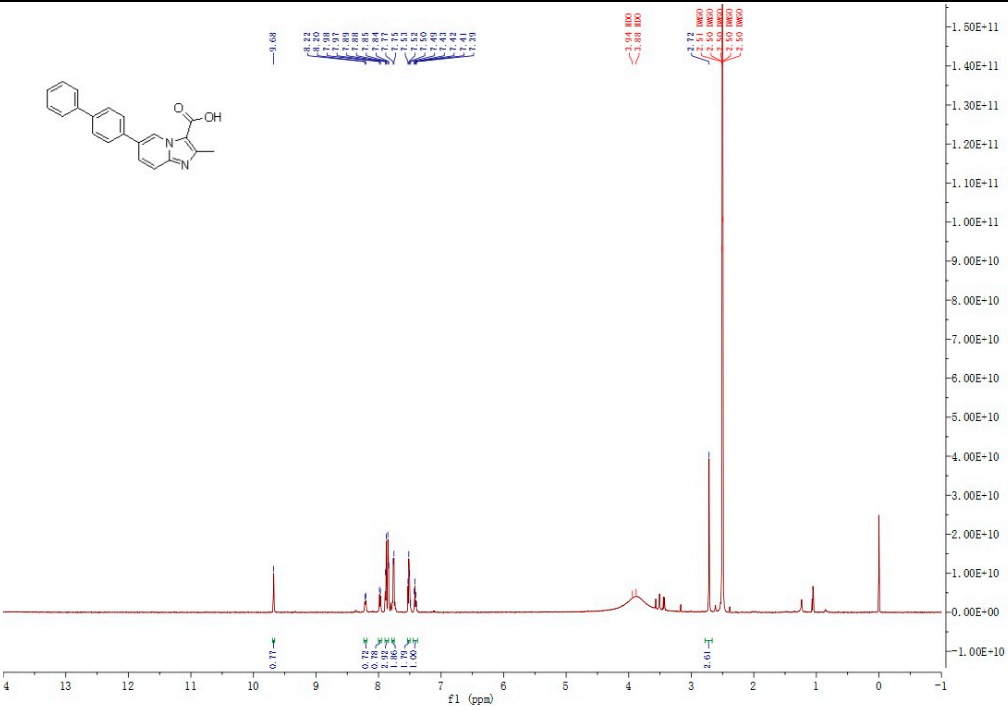
**^{13}C NMR**	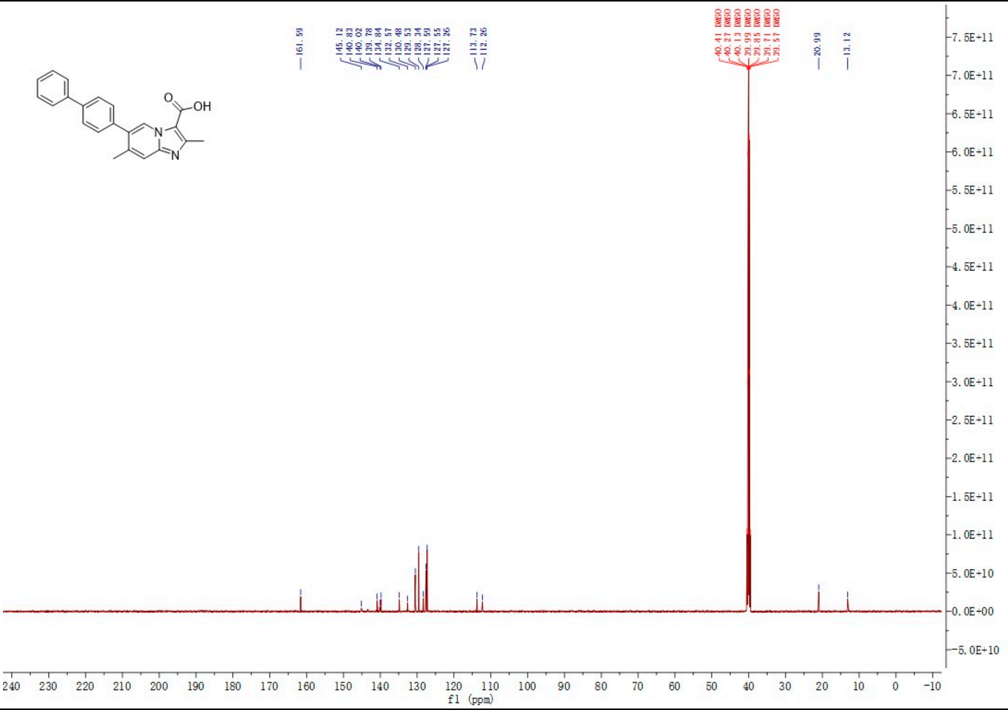
**Compound 12**	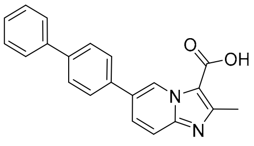

MS

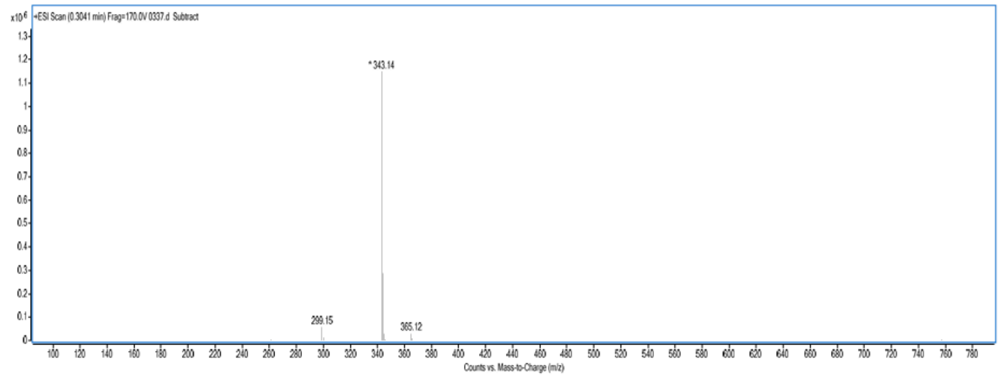

¹H NMR

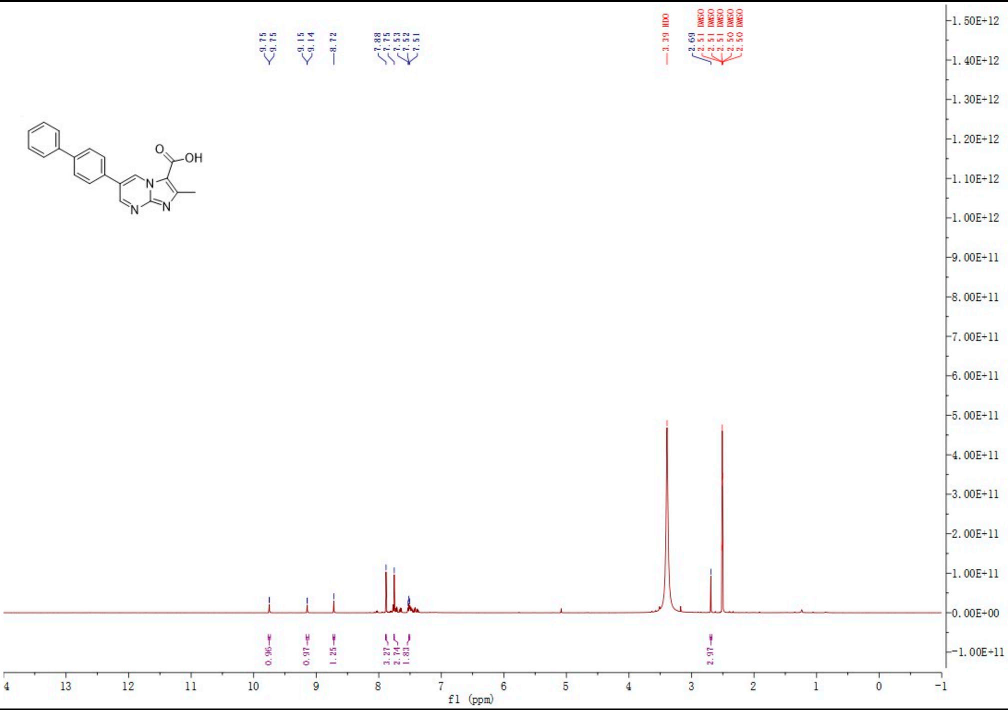

Compound 13

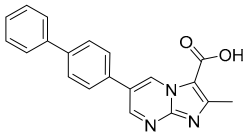

MS

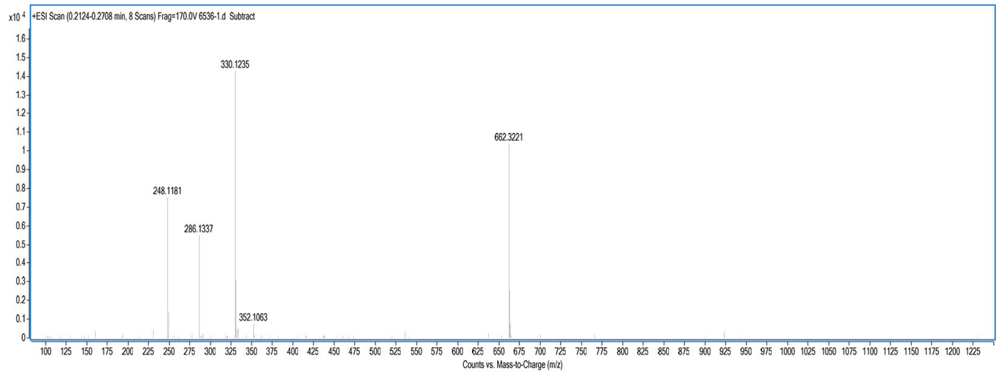

^{1}H NMR	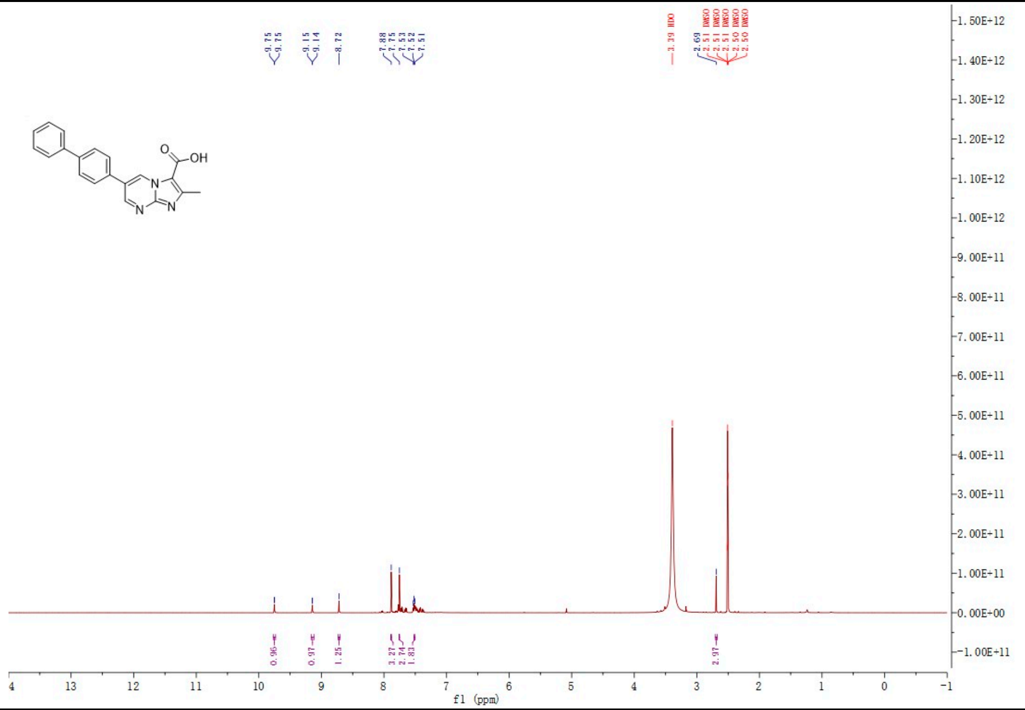
Compound 14	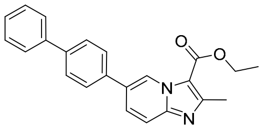
MS	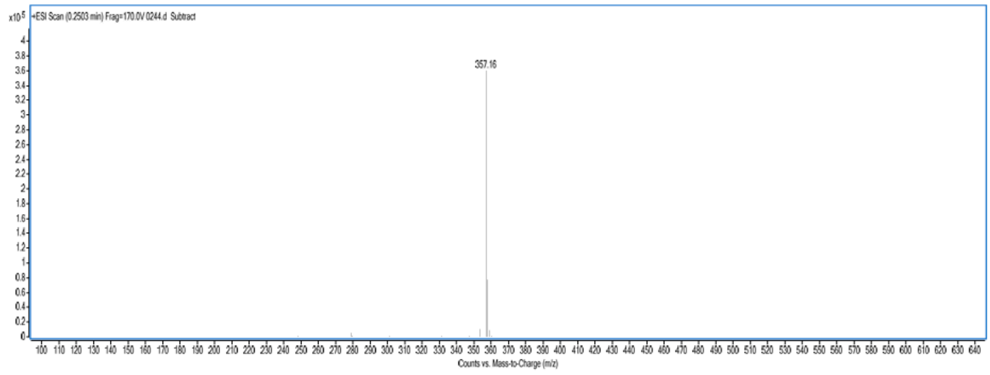

^{1}H NMR	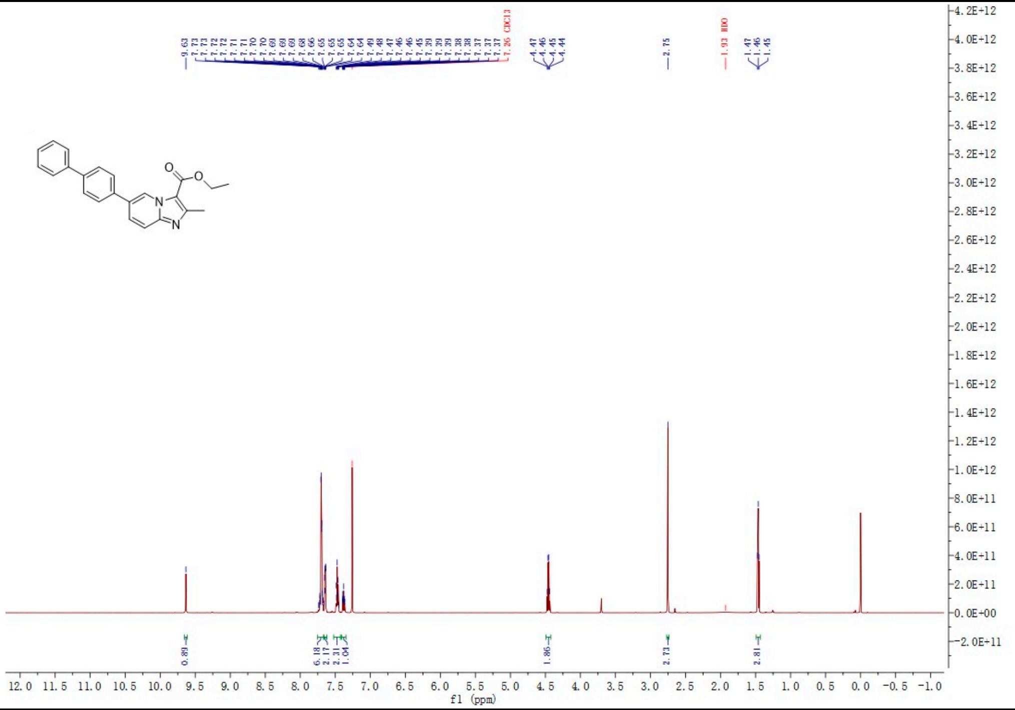
^{13}C NMR	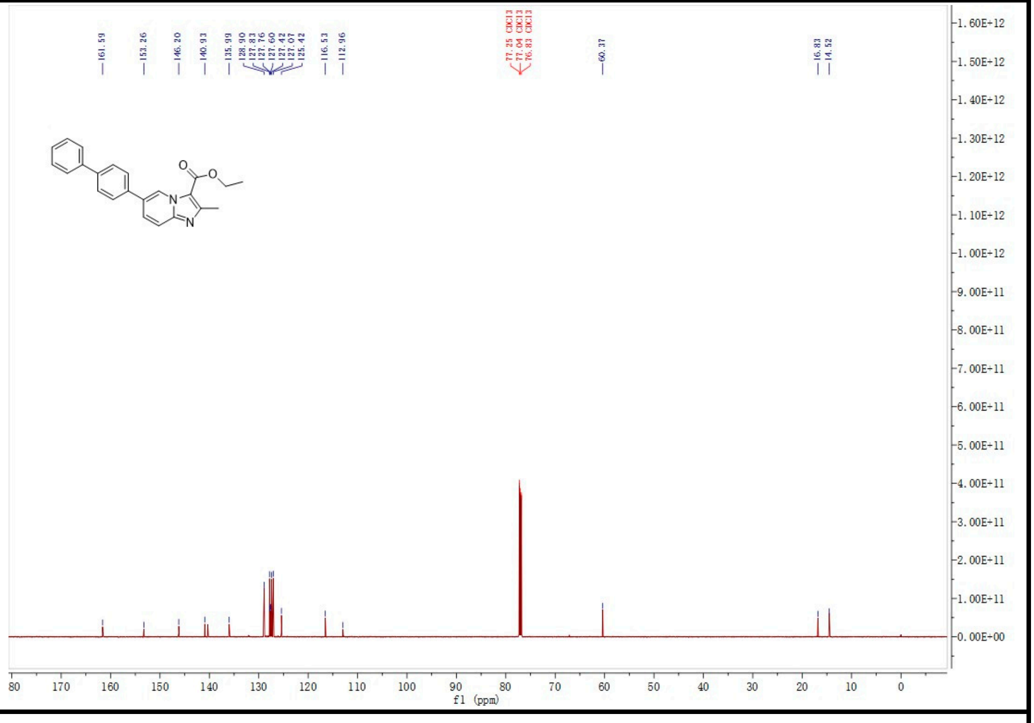
**Compound 15**	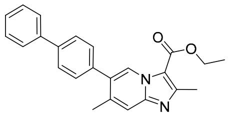

<table>
<tr><td>MS</td><td>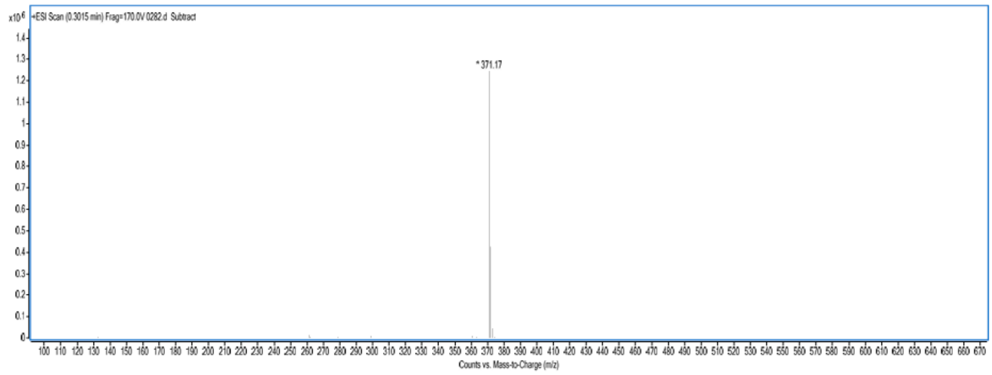
</td></tr>
<tr><td>¹H NMR</td><td>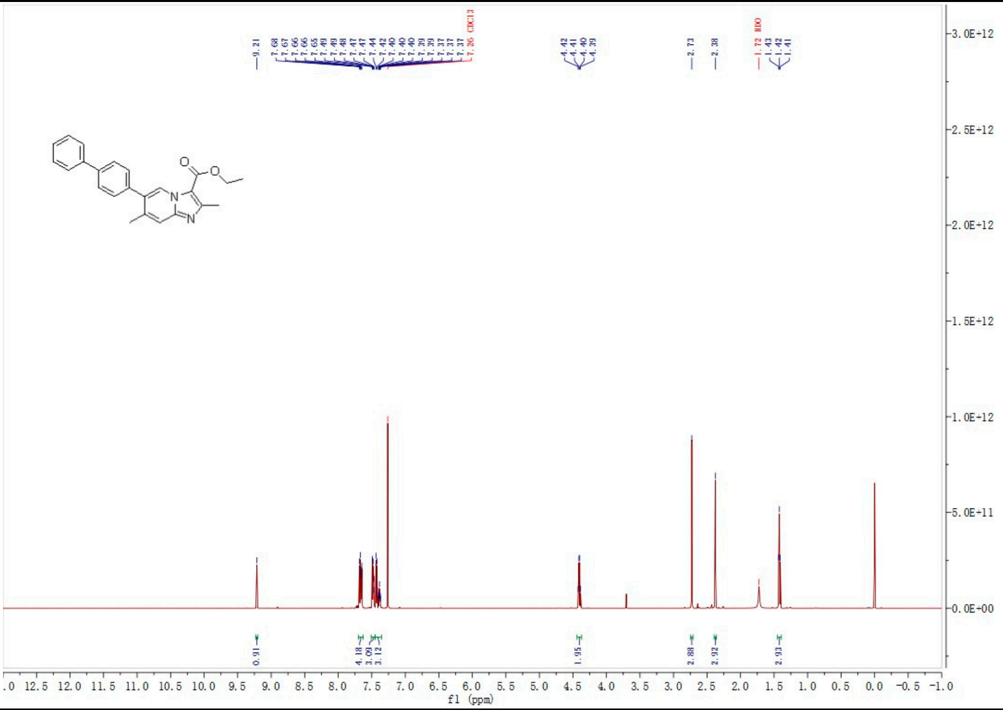
</td></tr>
<tr><td>¹³C NMR</td><td>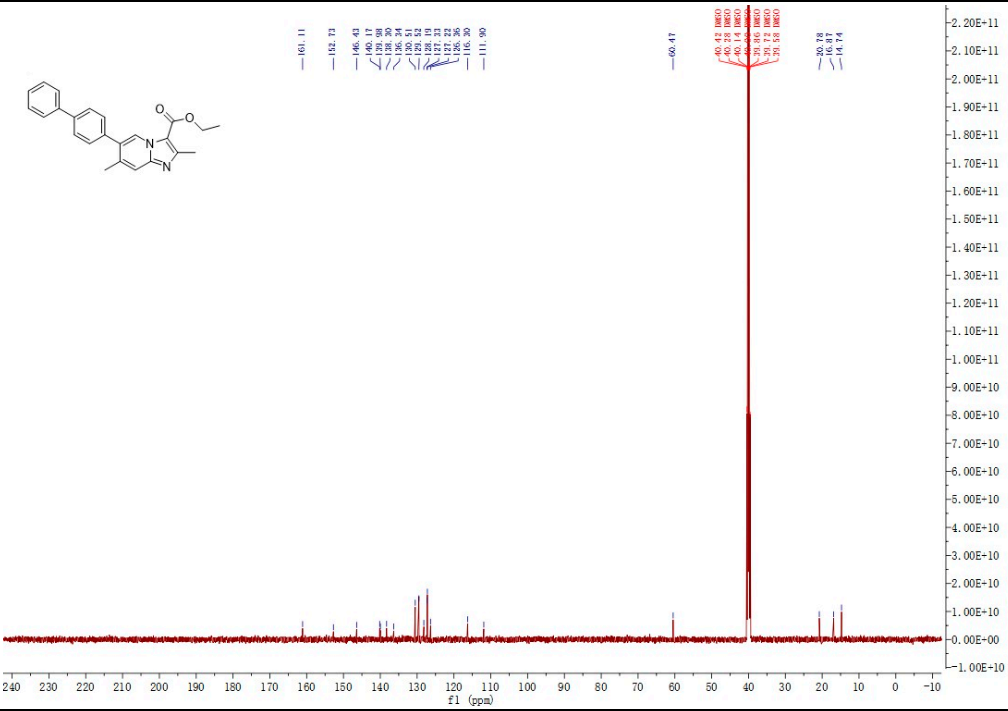
</td></tr>
</table>

**Compound 16**	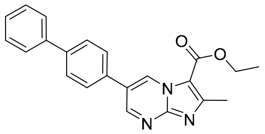
**MS**	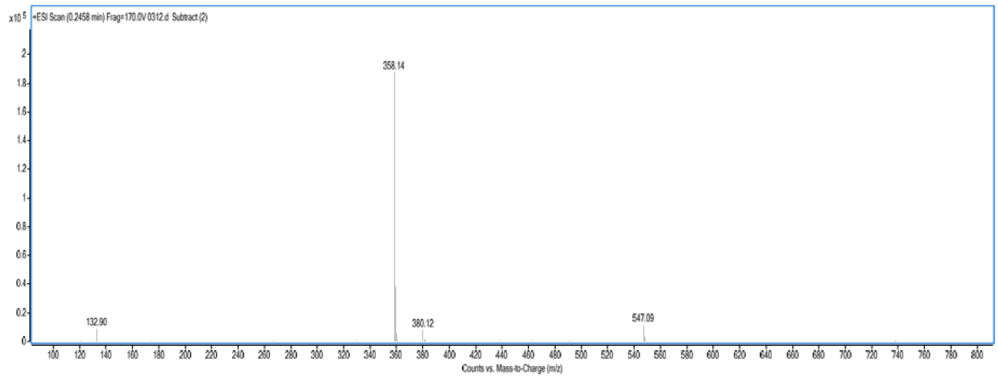
**^{1}H NMR**	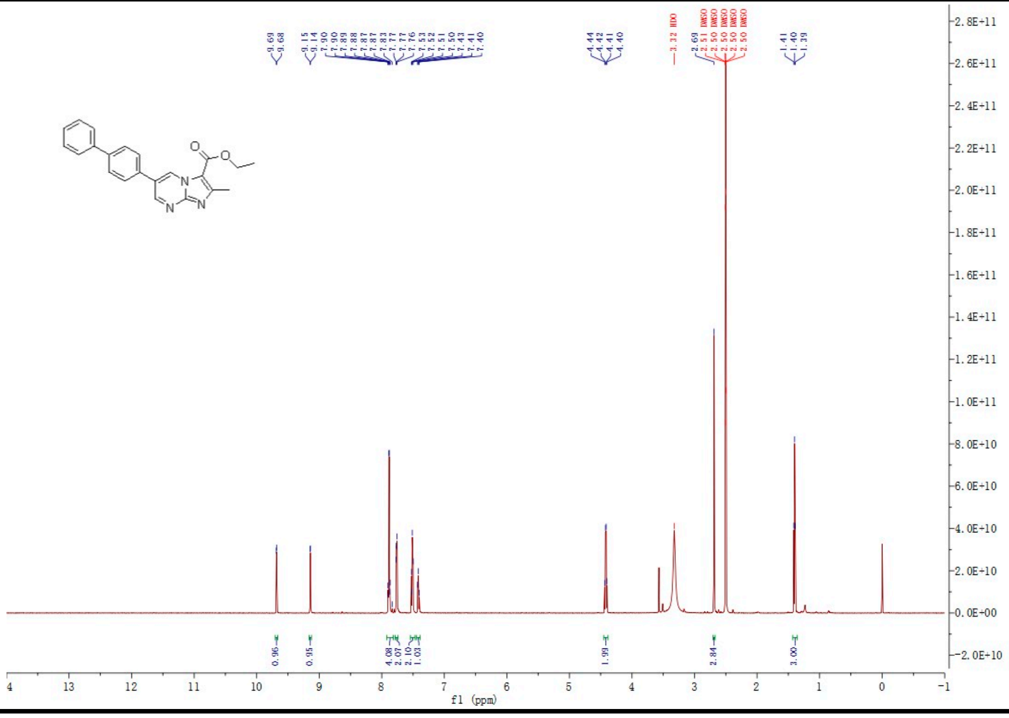

^{13}C NMR

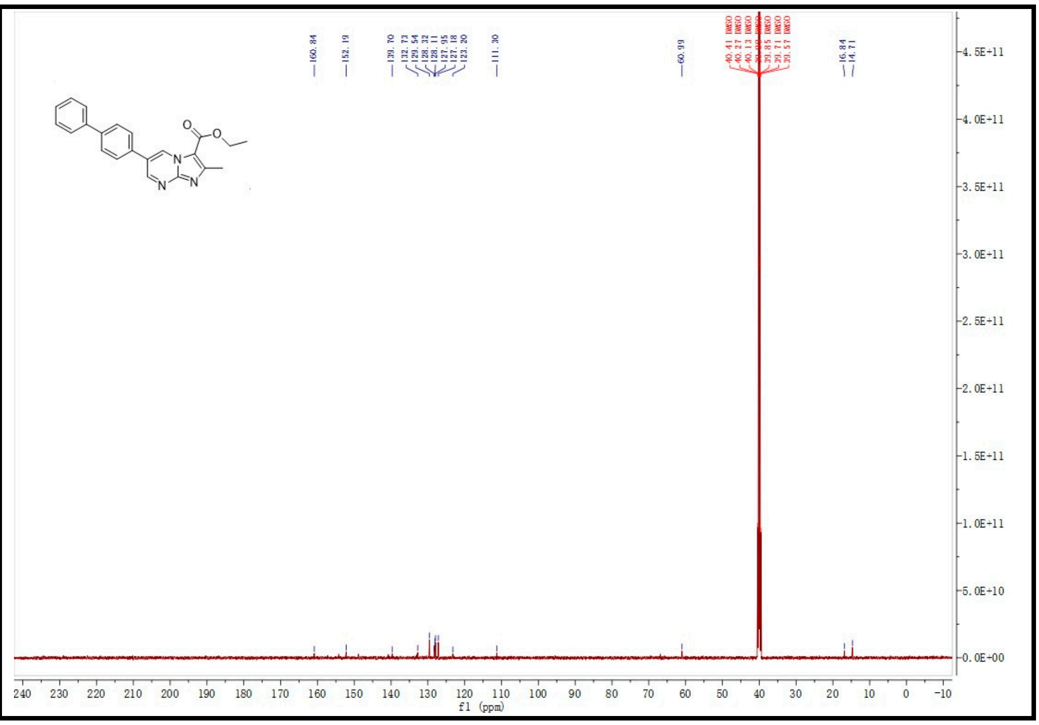

160.84
152.19
139.70
132.73
129.54
128.32
128.11
127.95
127.18
123.20
111.30
60.99
16.84
14.71
4.5E+11
4.0E+11
3.5E+11
3.0E+11
2.5E+11
2.0E+11
1.5E+11
1.0E+11
5.0E+10
0.0E+00
f1 (ppm)
